# Genome Mining and Evolutionary Analysis Reveal Diverse Type III Polyketide Synthase Pathways in Cyanobacteria

**DOI:** 10.1093/gbe/evab056

**Published:** 2021-03-19

**Authors:** Joachim Steen Larsen, Leanne Andrea Pearson, Brett Anthony Neilan

**Affiliations:** School of Environmental and Life Sciences, University of Newcastle, Newcastle, New South Wales, Australia

**Keywords:** type III PKS, evolution, cyanobacteria, (7.7)paracyclophane, hierridin, cytochrome b5

## Abstract

Cyanobacteria are prolific producers of natural products, including polyketides and hybrid compounds thereof. Type III polyketide synthases (PKSs) are of particular interest, due to their wide substrate specificity and simple reaction mechanism, compared with both type I and type II PKSs. Surprisingly, only two type III PKS products, hierridins, and (7.7)paracyclophanes, have been isolated from cyanobacteria. Here, we report the mining of 517 cyanobacterial genomes for type III PKS biosynthesis gene clusters. Approximately 17% of the genomes analyzed encoded one or more type III PKSs. Together with already characterized type III PKSs, the phylogeny of this group of enzymes was investigated. Our analysis showed that type III PKSs in cyanobacteria evolved into three major lineages, including enzymes associated with 1) (7.7)paracyclophane-like biosynthesis gene clusters, 2) hierridin-like biosynthesis gene clusters, and 3) cytochrome b5 genes. The evolutionary history of these enzymes is complex, with some sequences partitioning primarily according to speciation and others putatively according to their reaction type. Protein modeling showed that cyanobacterial type III PKSs generally have a smaller active site cavity (mean = 109.035 Å^3^) compared with enzymes from other organisms. The size of the active site did not correlate well with substrate size, however, the “Gatekeeper” amino acid residues within the active site were strongly correlated to enzyme phylogeny. Our study provides unprecedented insight into the distribution, diversity, and molecular evolution of cyanobacterial type III PKSs, which could facilitate the discovery, characterization, and exploitation of novel enzymes, biochemical pathways, and specialized metabolites from this biosynthetically talented clade of microorganisms.

**Significance**

Type III polyketide synthases (PKSs) are specialized biosynthetic enzymes found primarily in plants. Surprisingly, only two examples have been characterized from cyanobacteria (blue-green algae), which are prolific producers of polyketides and other natural products. This study demonstrated that type III PKSs are widespread in cyanobacteria and have evolved into three lineages, including one that is closely related to plant enzymes. The phylogeny of cyanobacterial type III PKSs, together with their putative structure and their association with diverse tailoring enzymes suggests that they are involved in the production of a wide range of type III polyketides.

## Introduction

Polyketide synthases (PKSs) are specialized enzymes that synthesize polyketides; a large group of natural products, including antibiotics, immunosuppressants, toxins, and a variety of other biologically active compounds (Jenke-Kodama et al. 2005; Shimizu et al. 2017). Cyanobacteria (blue-green algae are a particularly rich source of polyketides and hybrid compounds thereof, including microcystins, anatoxin, cylindrospermopsin, (7.7)paracyclophanes, and hierridins (Nakamura et al. 2012; Pearson et al. 2016; Costa et al. 2019). The native biological function of these compounds is yet to be resolved; however, they are believed to confer an ecological advantage to the producing strains. For example, the hybrid polyketide-non-ribosomal peptide toxins, the microcystins, are predicted to act as feeding deterrents, siderophores, signaling molecules, or antioxidants (Humble et al. 1997; Li et al. 2009; Zilliges et al. 2011; Gan et al. 2012).

**Fig. 1.**
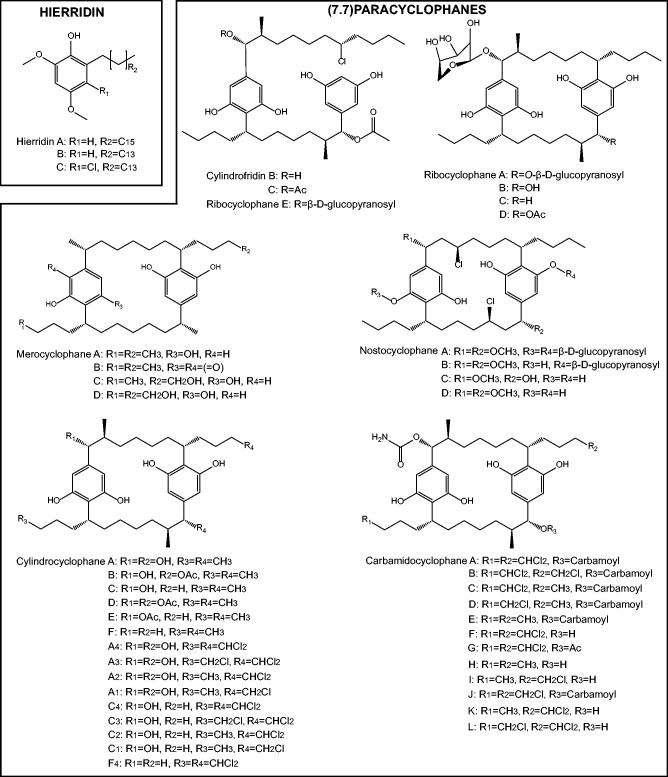
Compounds produced by cyanobacterial type III PKSs. Structures reproduced from Martins et al. (2019).

PKSs catalyze the decarboxylative Claisen condensation of malonyl-coenzyme A (CoA), methylmalonyl-CoA and/or ethylmalonyl-CoA units onto a substrate molecule forming polyketides in an analogous fashion to fatty acid synthesis (Lai and Cronan 2003) and are classified into three groups according to their structure. Type I PKSs are large, multi-modular enzymes widespread in bacteria (including cyanobacteria) that carry out condensation reactions in a stepwise manner. Type II PKSs are aggregates of monofunctional proteins, found mostly in actinobacteria, that carry out condensation reactions in an iterative way. Finally, type III PKSs are small homodimeric enzymes, found mostly in plants, which carry out condensation reactions in an iterative manner (Flores-Sanchez and Verpoorte 2009; Katsuyama and Ohnishi 2012). Type III PKSs are particularly interesting because of their relatively simple reaction mechanism and wide substrate specificity, giving rise to a diverse range of products (Katsuyama and Ohnishi 2012). Most polyketides identified in cyanobacteria, are produced by type I PKSs however, a select few, including the cytotoxic (7.7)paracyclophanes and antiplasmodial hierridins, are produced by type III PKSs (Bui et al. 2007; Costa et al. 2019) (fig. 1).

Cyanobacterial PKSs are encoded within large gene clusters (ranging from 3 to 60 kb in size), usually alongside genes for the modification and transport of the corresponding natural product. Occasionally, these gene clusters encode other biosynthetic enzymes, including fatty acid synthases (FAS) and non-ribosomal peptide synthetases (NRPS) (Tillett et al. 2000; Satou et al. 2013). In these cases, the different biosynthetic enzymes (FASs, PKSs, NRPSs etc.) work together to form a single product. Several such “hybrid” clusters have been identified in cyanobacteria, including the cyanotoxins microcystin and cylindrospermopsin, which are produced by both NRPSs and type I PKSs (Tillett et al. 2000; Mihali et al. 2008), and the alkylresorcinol dimer antibiotics, (7.7)paracyclophanes, which are produced by type I PKSs and type III PKSs (Nakamura et al. 2012). Hybrid type I and III polyketides are rare in nature, and the (7.7)paracyclophanes are the only such compounds to be isolated from cyanobacteria. Recently an analogue of these compounds, dedichloronostocyclophane D, was identified in extracts of the Vietnamese marine snail *Planaxis sulcatus* (Thanh et al. 2020). However, it is unclear whether it is produced by the snail, or a cyanobacterium occupying the same ecological niche.

In total, seven (7.7)paracyclophanes have been identified (fig. 1), however, only three of these have been associated with a gene cluster; the merocyclophanes (May et al. 2017), the carbamidocyclophanes (Preisitsch, Heiden, et al. 2016), and the cylindrocyclophanes (Nakamura et al. 2012). All share the same skeleton, consisting of two *p*-substituted aromatic rings (resorcinols) bridged between two C_12_ ester chains. The biosynthesis of cylindrocyclophanes, the best characterized (7.7)paracyclophane, starts with the attachment of a decanoic acid (C_10_ ester) to a free-standing acyl carrier protein (ACP) unit (CylB). The decanoyl-ACP thioester is putatively chlorinated by a novel halogenase (CylC) (Nakamura et al. 2017) and elongated by two type I PKSs (CylD and CylH). The ketide chain is then transferred to a type III PKS (CylI), which carries out the final elongations and cyclization of the aromatic ring, giving rise to an alkyl resorcinol intermediate. An alkylating enzyme (CylK) then dimerizes two alkyl resorcinol intermediates via a head-to-tail mechanism, substituting the chlorination with the resorcinol group from the other intermediate to form the final (7.7)paracyclophane (Nakamura et al. 2012, 2017). Linear compounds known as cylindrofridins (fig. 1) may be produced in the absence of CylC or CylK (Preisitsch, Niedermeyer, et al. 2016).

Until recently, it was believed that type III PKSs only used CoA-activated substrates, unlike type I and type II PKSs, which use ACP-bound substrates. However, recent studies have demonstrated that some type III PKSs, including CylI, can use both CoA- and ACP-bound substrates (Chemler et al. 2012; Nakamura et al. 2012; Preisitsch, Heiden, et al. 2016; May et al. 2017). As only a few examples have been enzymatically characterized, it is unclear whether this relaxed substrate specificity is a common feature of type III PKSs.

Another family of compounds synthesized by type III PKSs in cyanobacteria is the hierridins, O-methylated monoalkylresorcinols featuring a long-aliphatic chain (fig. 1). First isolated from *Phormidium ectocarpi* in 1998, hierridins were shown to have antiplasmodial and antitumor activity (Papendorf et al. 1998; Leão et al. 2013). Unlike the (7.7)paracyclophanes, the proposed biosynthesis of hierridins occurs through a single-type III PKS (HidC) (Costa et al. 2019). Starting with a C_14_ acyl-ACP thioester, HidC carries out three elongations with malonyl-CoA before release and cyclization of the resorcinol ring. Thereafter, the structure undergoes reduction and methylation (by HidA and HidB), forming hierridin B. Chlorination of the ring by a yet unknown mechanism gives rise to hierridin C.

The functional diversity of type III PKSs and their relatively sparse distribution in plants, fungi, and bacteria compared with type I and II PKSs, make them an interesting group of enzymes to investigate from both an evolutionary and biosynthetic perspective. Here, we mined publicly available cyanobacterial genomes for type III PKS gene clusters and examined their composition, arrangement, and conservation. We also performed a detailed phylogenetic analysis of the encoded type III PKSs and modeled representative enzymes from each clade. The results are discussed in terms of the distribution, diversity, evolution, and putative function of these under-explored biosynthesis pathways.

## Materials and Methods

### Genome Mining and Gene Cluster Comparisons

Cyanobacterial genomes were downloaded from GenBank (National Centre for Biotechnology Information, NCBI) and mined for type III PKS biosynthesis gene clusters using both the Antibiotics and Secondary Metabolite Analysis Shell (antiSMASH version 5.1) set to default (relaxed strictness) with all extra features enabled (Blin et al. 2019) and Basic Alignment Search Tool (BLAST, NCBI) set to default with a cut-off *E*-value of 10^−20^. Query sequences used for BLAST were ArsB (ZP_00418325), CabI (AMB48450), CHS (P30075), CylI (AFV96143), HidC (QBC65480), MerE (AQA28564), ORAS (EAA31191), PKS18 (P9WPF3), THNS (Q9FCA7), and TotC1 (ATL73040). Because the quality of the prediction by antiSMASH is highly dependent on the quality of the input data (i.e., gene clusters may not be detected if they are scattered over multiple small contigs), only RefSeq genomes were used.

Mined and reference gene clusters were organized into a similarity network using the Biosynthetic Genes Similarity Clustering and Prospecting Engine (BIG-SCAPE) (Navarro-Munoz et al. 2020) and compared using Clinker v 0.0.12 (Gilchrist and Chooi 2021).

Type III PKS genes and their inferred primary peptide sequences were extracted from the mined gene clusters for subsequent phylogenetic analysis and protein modeling. Previously characterized type III PKSs (reference sequences) were identified through a literature search and downloaded from GenBank (supplementary table 1, Supplementary Material online). 16 s rRNA gene sequences from the same species were also extracted from the cyanobacterial genomes using RNAmmer version 1.2 (Lagesen et al. 2007). In cases where RNAmmer could not identify 16 s rRNA gene sequences, they were downloaded from the SILVA database (https://www.arb-silva.de/search/, last accessed September 17, 2020) (Yilmaz et al. 2014).

### Phylogenetic Tree Construction

A multiple sequence alignment of putative type III PKS peptide sequences and reference sequences was performed using MUSCLE (Edgar 2004) with default settings. The alignment was trimmed to remove large gaps (final length = 334 amino acids). A phylogenetic tree based on the alignment was constructed using MrBayes (Huelsenbeck and Ronquist 2001; Ronquist et al. 2012) with default settings and the best fitting model, LG + I + G, which was identified by Prottest3.2. MrBayes was run until the standard deviations were below 0.01.

The 16 s rRNA gene phylogenetic analysis was performed as above, with the following exceptions: DNA sequences were aligned using ClustalW (Thompson et al. 1994), with default settings. The alignment was trimmed to remove large gaps (final length = 775 nucleotides). The tree was constructed using MrBayes with default settings and the best fitting model, JC, which was identified by JModelTest2.1.10. MrBayes was run until the standard deviations were below 0.01.

### Protein Modeling

One cyanobacterial enzyme from each of the clades identified in the phylogenetic study was modeled using the online software, I-TASSER (Yang et al. 2015). The model with the highest Template modeling (TM) score was chosen for further analysis. The TM-Score is a means to measure how good the model is based on a given crystal structure; the higher the score, the better the model (Zhang and Skolnick 2004). The models were compared with published crystal structures for type III PKSs found in the Protein Data Bank (PDB) (supplementary table 4, Supplementary Material online). Models were visualized using PyMol2.0. The active site sizes of all the investigated enzymes were predicted using the online software CASTp3.0 with default settings (http://sts.bioe.uic.edu/castp, last accessed September 11, 2020).

## Results and Discussion

### Distribution and Diversity of Cyanobacterial Type III PKSs

**Fig. 2.**
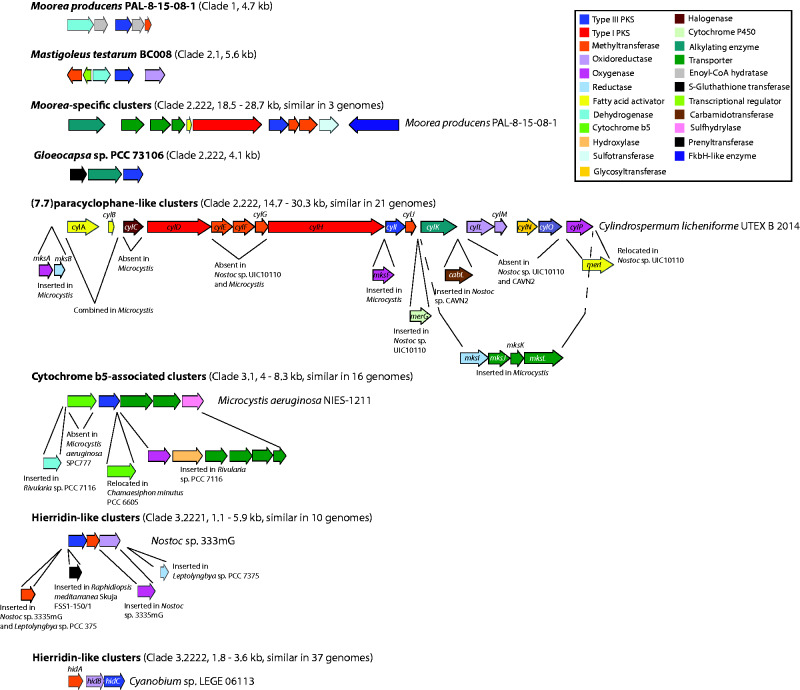
Distribution and diversity of type III PKS gene clusters from cyanobacteria. Clade numbers correspond to the type III PKS phylogenetic tree (fig. 3). Detailed comparison of gene cluster families using Clinker v 0.0.12 can be found in the Supplementary Material online.

To better understand the distribution and diversity of type III PKSs pathways in cyanobacteria, 517 cyanobacterial RefSeq genomes were downloaded from GenBank and mined using antiSMASH (Blin et al. 2019) and BLAST. Both methods returned the same results. Eighty-seven of the genomes analyzed (∼16.8%) harbored one or more type III PKS gene cluster. Genomes of *Moorea producens* PAL-8-15-08-1, *Nostoc* sp. 3335mG, *Cyanobium gracile* PCC 6307, and *Microcystis aeruginosa* PCC 7005 harbored two type III PKS gene clusters, taking the total number of identified cyanobacterial type III PKS gene clusters to 91 (results summarized in supplementary table 2, Supplementary Material online). By comparison, 34% of fungal genomes (Navarro-Muñoz and Collemare 2020) and 100% of plant genomes (Austin and Noel 2003) encode type III PKS pathways. Sequence similarity networks grouped the clusters into 60 families, where 49 were singletons (i.e., single cluster families). Clusters from *Prochlorococcus* and *Microcystis* formed the two largest families comprising 10 and 11 members, respectively (supplementary fig. 1, Supplementary Material online). Representative type III PKS gene clusters are depicted in figure 2.

### Hybrid Type I/Type III PKS Gene Clusters

Twenty-one (7.7)paracyclophanes-like biosynthesis gene clusters were identified in the cyanobacterial genomes analyzed. (7.7)paracyclophanes are the best-studied type III PKS metabolites from cyanobacteria. As mentioned above, they are synthesized through an unusual hybrid type I/III PKS system, which has not been identified in other phyla. To elucidate the genetic basis for (7.7)paracyclophanes diversity, we compared the mined gene clusters, including those responsible for the biosynthesis of carbamidocyclophane (*Nostoc* sp. CAVN2), cylindocyclophane (*Cylindrospermum lichenforme* UTEX B 2014), and merocyclophane (*Nostoc* sp. UIC10110), and the orphan type I/III PKS gene cluster from *M. aeruginosa* PCC 7806 (MKS, “*Microcystis* Ketide Synthase”). Although no metabolite has been matched to the MKS cluster, its transcription appears to be inversely co-regulated with the production of the hepatotoxin microcystin, making this gene cluster of special interest (Makower et al. 2015).

The (7.7)paracyclophane-like biosynthesis gene clusters display similar overall composition and organization (fig. 2). The carbamidocyclophane (*cab*) and cylindrocyclophane (*cyl*) biosynthesis gene clusters share particularly high sequence homology (∼80% identity). The merocyclophane (*mer*) biosynthesis cluster differs with regard to the type I PKS (*merC*) module organization, having unique dehydrogenase (DH) and enoylreductase (ER) domains. The Type I PKS, *merD*, also has a thioesterase (TE) in place of the terminal ACP found in *cabD* and *cylD*. However, the main biosynthesis genes, including the type III PKS (*merE*), are otherwise well conserved. In contrast, the genes encoding tailoring enzymes vary from cluster to cluster, indicating horizontal gene transfer or gene loss has shaped these clusters. For instance, the carbamidotransferase gene (*cabL*), which carries out the attachment of the carbamido group to the final carbamidocyclophane is unique to the carbamidocyclophane biosynthesis cluster, whereas the two oxidoreductase genes (*cylL* and *cylM*) might be important for the production of cylindrocyclophane D and E (fig. 1). The *cyl* cluster also contains a glycosyltransferase gene homologue (*cylN*), putatively involved in the production of ribocyclophanes; glycosylated analogous of cylindrocyclophanes (May et al. 2018). Strikingly, the AMP-binding enzyme (*cabA/cylA/merI*) is differentially located in the merocyclophane biosynthesis cluster compared with the cylindrocyclophane and carbamidocyclophane biosynthesis clusters, indicative of a genomic rearrangement in the former. Quite strikingly, the *mer* cluster also has fewer tailoring enzymes than the other (7.7)paracylophane biosynthesis clusters. This suggests that a deletion at the 3ʹ end might have occurred, resulting in the differential location of the AMP-binding enzyme.

The MKS cluster is more divergent from the characterized (7.7)paracyclophane biosynthesis gene clusters than they are from each other. This could reflect taxonomic divergence between *Microcystis* and the *Nostoc/Cylindrospermum* lineage, and the possible production of different type III PKS products in the *Microcystis* genus. However, parts of the second type I PKS gene (*mksE*), the type III PKS gene (*mksG*), and the upstream methyltransferase (*mksH*) are well conserved in the *Microcystis* and *Nostoc/Cylindrospermum* lineages. Interestingly, *mksC* encodes an AMP-binding enzyme (homologue of *cabA*, *cylA*, and *merA*) together with an ACP domain (homologue of the free-standing ACP encoded by *cabB*, *cylB*, and *merA*). This suggests that there has been a recombination event between the AMP-binding enzyme and the free-standing ACP in *mksC*. Equally interesting is the absence of *cylC* (halogenase) and *cylK* (alkylating enzyme) homologues in the MKS gene cluster, which are highly conserved in the other non-*Microcystis* clusters. CylC and CylK are important for the dimerization of the final ketide chain in the biosynthesis of (7.7)paracyclophane to form the characteristic cyclic structure. The absence of these two genes in the MKS cluster suggests that it does not produce a dimeric, cyclic structure-like (7.7)paracyclophane, but perhaps a linear analogue. Interestingly, the *Microcystis* clusters all contain three transporters, two encoding resistance nodulation cell division (RND) family transporters and one encoding an major facilitator superfamily (MFS) transporter. This suggests that the metabolites produced by these clusters have an extracellular function. As no type III PKS products have been isolated and identified from *Microcystis*, further research is needed to verify the compound produced by the MKS cluster. All the MKS clusters from *Microcystis* have the same composition and structural organization.

Detailed comparison of (7.7)paracyclophane-like biosynthesis gene cluster families using Clinker v 0.0.12 can be found in supplementary figure 2, Supplementary Material online.

### Hierridin-Like Biosynthesis Gene Clusters

Forty-seven hierridin-like biosynthesis gene clusters were identified in the cyanobacterial genomes analyzed (fig. 2). As mentioned above, hierridins are synthesized by a single type III PKS. The cyclized polyketide is then modified by a methyltransferase and an oxidoreductase to produce herridin B. In *Cyanobium* sp. LEGE 06113, the hierridin skeleton is chlorinated by an unknown mechanism to produce hierridin C (fig. 1) (Costa et al. 2019). So far, this gene cluster is the only of its class to have been associated with a compound, however, as we have shown, multiple hierridin-like biosynthesis gene clusters are present in cyanobacteria, particularly in the *Prochlorococcus* and *Synechococcus* genera, suggesting that production of hierridins and related compounds is common in marine species (supplementary table 2, Supplementary Material online).

Compared with the (7.7)paracyclophane-like biosynthesis gene clusters, the hierridin-like clusters are mostly well conserved in terms of composition and organization (fig. 2). The major difference being two different patterns of gene arrangement: *hidA>hidB*, *>hidC* and *hidC>hidA>hidB*, with the former arrangement being the more conserved. Besides encoding HidA (methyltransferase), HidB (oxidoreductase), and HidC (type III PKS), the hierridin-like biosynthesis gene clusters encode a variety of potential stand-alone tailoring enzymes. For instance, the cluster from *Leptolyngbya* sp. PCC 7375 encodes a second methyltransferase and a reductase, suggesting that this species produces a poly-methylated compound, which has been reduced further than hierridin B.

Two hierridin-like biosynthesis gene clusters were identified in the genome of *Nostoc* sp. 335 mg. One of the clusters encodes two methyltransferases and an oxygenase, but lacks the oxidoreductase, suggesting a deletion might have occurred. The other cluster encodes the three main biosynthesis genes but no additional tailoring enzymes. The presence of two gene clusters in *Nostoc* sp. 335 mg suggests that duplication and deletion events occurred, resulting in the duplication of the methyltransferase and loss of the oxidoreductase. Uniquely, the hierridin-like biosynthesis gene cluster from *Raphidiopsis mediterranea* Skuja FSS1-150/1 encodes a prenyltransferase, suggesting that this species produces a novel prenylated compound.

Detailed comparison of hierridin-like biosynthesis gene cluster families using Clinker v 0.0.12 can be found in supplementary figure 3, Supplementary Material online.

### Gene Clusters Encoding Type III PKSs and Cytochrome b5

Sixteen gene clusters encoding type III PKS and cytochrome b5 genes were identified in the genomes of *Nostocales* and *Chroococcales* species (fig. 2). These gene clusters were poorly conserved in terms of sequence homology and structural organization, compared with the (7.7)paracyclophane and hierridin-like biosynthesis gene clusters.

Gene clusters encoding type III PKSs and cytochrome genes (cytochrome P450 or cytochrome b5) have previously been identified in mycobacteria. In *M. tuberculosis* H37Rv, the encoded type III PKS (PKS18) is an alkylpyrone synthase, which uses long aliphatic acids as substrates (C_6_–C_20_), extending them two or three times to form triketide and tetraketide pyrones, respectively (Saxena et al. 2003). Although the activity of the associated cytochrome b5 is unclear, it is likely involved in the oxidation of the type III PKS product, giving rise to myolic acid, a membrane lipid found in mycobacteria (Kolattukudy et al. 1997; Saxena et al. 2003). In the cyanobacterial genomes investigated here, cytochrome b5 is encoded just upstream of the type III PKS genes (or downstream in the case of *Chamaesiphon minutus* PCC 6605), suggesting a role in biosynthesis. As no cyanobacterial cytochrome b5-associated gene clusters have been experimentally characterized, their products remain unknown. However, it seems unlikely that they are involved in the synthesis of myolic acid, as in *M. tuberculosis*, as this compound has not been isolated from any cyanobacterial species (Rajeshwari and Rajashekhar 2011).

The cyanobacterial type III PKS gene clusters associated with cytochrome b5 also encode a combination of the three transporter families; RND, MFS, and ATP-binding cassette (ABC) transporters, whereas only RND and MFS transporters are present in the *Microcystis* (7.7)paracyclophane-like biosynthesis gene clusters. Interestingly, the cluster from *Rivularia* sp. PCC 7116 encodes all families, whereas the cluster from *Microcystis aeruginosa* SPC777 lacks the MFS transporter, indicating a deletion in this strain. The presence of the transporters suggests that the produced compounds have an extracellular role. In contrast, the *M. tuberculosis* H37Rv cluster does not encode a transporter.

Interestingly, the gene cluster from *Rivularia* sp. PCC 7116 encodes a stand-alone hydroxylase. To our knowledge, only one other stand-alone hydroxylase gene (*srsC*) has been associated with a type III PKS cluster (the *srsABC* cluster from *S. griseus* NBRC 13350, involved in the production of alkylresorcinols) (Funabashi et al. 2008). However, hydroxylation of type II PKS products (after the extension step) is relatively common (Peric-Concha et al. 2005; Teufel et al. 2013). Likewise, the *M. aeruginosa* SPC777 and NIES-1211 gene clusters encode a sulfhydrylase, suggesting that sulfonation occurs at some point during biosynthesis or chain termination, as in the curacin A pathway (Gu et al. 2009). Alternatively, this enzyme could act as a transacylase-like Fub7 from the fusaric acid (type I PKS) pathway in *Fusarium* species (Brown et al. 2015; Hai et al. 2020). Fub7 formally converts *O*-acetyl-l-homoserine into corresponding *O*-acyl-l-homoserines however it can also synthesize L-homocysteine in the presence of hydrogen sulfide (Hai et al. 2020).

Detailed comparison of cytochrome b5-associated type III PKS gene cluster families using Clinker v 0.0.12 can be found in supplementary figure 4, Supplementary Material online.

### Phylogeny of Cyanobacterial Type III PKSs

To understand the evolution and predict the activity of type III PKSs from cyanobacteria, a phylogenetic analysis was conducted based on the inferred primary peptide sequences of mined type III PKS genes and previously characterized cyanobacterial enzymes, including CylI (cylindrocyclophane synthase) from *Cylindrospermum licheniforme* UTEX B 2014 (Nakamura et al. 2012), MerE (merocyclophane synthase) from *Nostoc* sp. UIC 10110 (May et al. 2017), CabI (carbamidocyclophane synthase) from *Nostoc* sp. CAVN2 (Preisitsch, Heiden, et al. 2016), and HidC (hierridin synthase) from *Cyanobium* sp. LEGE 06113 (Costa et al. 2019). Other type III PKS reference sequences from plants, bacteria and fungi were also included in the analysis. The FAS, FabH, from *E. coli* was used as the outgroup (supplementary table 1, Supplementary Material online). Parts of the multiple sequence alignment upon which the tree was based are shown in supplementary material (supplementary fig. 5, Supplementary Material online). A second phylogenetic analysis based on the 16S rRNA genes of the same species was also conducted to infer speciation (supplementary fig. 6, Supplementary Material online).

**Fig. 3.**
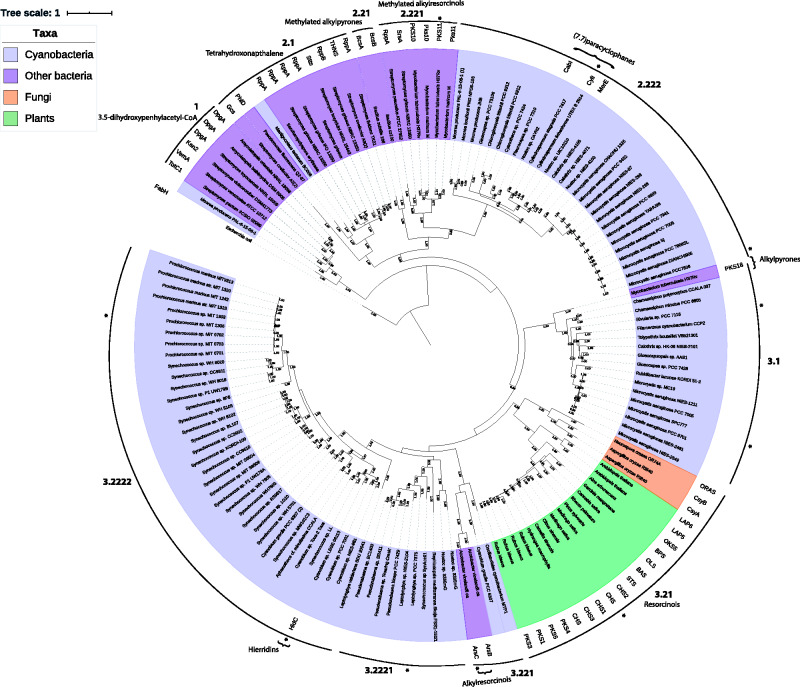
Phylogenetic tree of type III PKSs found in cyanobacteria, other bacteria, plants, and fungi. The tree is based on the inferred primary peptide sequences of 142 type III PKSs. Clades are numbered in a hierarchical manner. Names of characterized enzymes are provided next to the species name. The tree is color-coded according to speciation; cyanobacteria (light blue), bacteria (purple), plants (green), and fungi (orange). The most common product for each characterized enzyme/family is indicated. Sequences used for protein modeling are indicated by an asterisk. The tree was generated using MrBayes with the LG + I + G model. Bootstrap values from 1,000,000 replicates above a threshold of 0.9 are represented on branches. The scale bar represents one amino acid substitution per amino acid site.

The type III PKS sequences partitioned into three major clades: two bacterial clades (Clades 1 and 2) and a clade comprising mostly eukaryotic and cyanobacterial sequences (Clade 3) (fig. 3). Our overall tree is largely in agreement with a former study by Shimizu et al. (2017), which demonstrated that the evolution of type III PKS sequences is complex, with some sequences partitioning primarily according to speciation (analogous to 16S rRNA gene phylogeny, supplementary fig. 6, Supplementary Material online) and others putatively according to their reaction type (supplementary table 3, Supplementary Material online).

Clade 1 comprised actinobacterial type III PKSs (e.g., DpgA, Ken2, VemA) encoded within hybrid gene clusters (fig. 3), involved in the production of (3,5-dihydroxyphenyl)acetyl-CoA, a precursor of glycopeptide antibiotics. Enzymes within this clade initiate biosynthesis of the ketide chain, via condensation of four malonyl-CoAs, before transfer to two type I PKSs for further elongation. Cyclization occurs via a C8–C3 Claisen reaction (Wu et al. 2012; Zeng et al. 2012; Chen et al. 2017) (supplementary table 3, Supplementary Material online). A single cyanobacterial type III PKS from *Moorea producens* PAL-8-15-08-01 was present within this clade, suggesting that this species is capable of producing glycopeptide antibiotics or similar compounds. However, the gene cluster from this species is not similar to any of the other bacterial gene clusters that partitioned in this clade (fig. 2).

Clade 2 comprised a variety of bacterial and cyanobacterial sequences, which partitioned further into two subclades: Clades 2.1 and 2.2. Clade 2.1 comprised actinobacterial enzymes (e.g., RppA, StTS, THNS) involved in tetrahydroxynaphthalene production, a precursor of flaviolin. Enzymes in this clade almost exclusively use malonyl-CoA starter substrates and C3–C8 or C2–C7 aldol condensation cyclization mechanisms, the only exception being Gcs, which uses either methylmalonyl-CoA or ethylmalonyl-CoA before carrying out a lactonization to produce germicidin (Chemler et al. 2012) (supplementary table 3, Supplementary Material online).

Clade 2.2 was divided into two subclades: Clade 2.21 and 2.22. Clade 2.21 comprised *Bacillus* enzymes (e.g., BcsA and BcsB) involved in the production of long aliphatic tri- and tetraketide pyrones. The pyrones are produced by condensation of a long aliphatic thioester with either malonyl- or methylmalonyl-CoA. This is followed by a lactonization to form the final pyrone (Nakano et al. 2009). Clade 2.22 comprised characterized enzymes from actinobacteria (e.g., SrsA, PKS10, PKS11) and cyanobacteria (e.g., CabI, CylI, MerE) involved in the synthesis of alkylresorcinols (supplementary table 3, Supplementary Material online), as well as 25 uncharacterized enzymes from cyanobacteria. Within Clade 2.22, actinobacterial and cyanobacterial sequences formed distinct monophyletic groups. The actinobacterial enzymes are known to use long aliphatic esters as substrates and carry out either a lactonization or C2–C7 aldol condensation with either malonyl- or methylmalonyl-CoA (Saxena et al. 2003; Sirakova et al. 2003). The only exception is RppA, which is similar to the enzymes found in Clade 2.1, in terms of its substrate and extender specificities (Ghimire et al. 2008). The previously characterized cyanobacterial enzymes in Clade 2.22 use malonyl-CoA to extend dodecanoyl-CoA (C_12_ thioester obtained from a type I PKS) and cyclize it via a C2–C7 aldol condensation (Kang et al. 2012; Nakamura et al. 2012; Luo et al. 2014). Significantly, nearly all the cyanobacterial sequences in Clade 2.22 originate from hybrid clusters containing genes encoding both type I and type III PKSs (fig. 2), where the type III PKS carries out the last reaction in the corresponding pathway. The exception is the *Gloeocapsa* sp. PCC 73106 enzyme, which was mined from a cluster containing a single type III PKS. None of the characterized actinobacterial enzymes from clade 2.1 or clade 2.22 originate from hybrid clusters. The enzymes mined from *Microcystis* (including MksG from the MKS cluster) formed a monophyletic group, and their associated biosynthesis gene clusters displayed a different architecture to those encoding the other cyanobacterial type III PKSs in Clade 2.22, that is, they lacked genes encoding halogenase and alkylating enzymes (*cylC* and *cylK* homologues) (Nakamura et al. 2017) (fig. 2). This double gene deletion in *Microcystis* could foreseeably enable the production of a novel monomeric polyketide similar to the cylindrofridins (fig. 1) (Preisitsch, Niedermeyer, et al. 2016).

Clade 3 was the largest and most diverse clade in the tree, comprising mainly eukaryotic and cyanobacterial type III PKSs. The clade partitioned into two subclades: Clade 3.1 and 3.2. Clade 3.1 comprised uncharacterized type III PKSs encoded alongside cytochrome b5 (fig. 2). Except for the alkylpyrone biosynthesising PKS18 from *Mycobacterium tuberculosis*, all sequences within this subclade originated from cyanobacteria. PKS18 uses long aliphatic esters as substrates and extends those with malonyl-CoA, followed by a lactonization to form the final pyrones (Saxena et al. 2003; Sankaranarayanan et al. 2004) (supplementary table 3, Supplementary Material online). The uncharacterized cyanobacterial enzymes in this clade are likely to incorporate similar substrates and reaction mechanisms. Interestingly, no alkylpyrones have been isolated from cyanobacteria; all type III PKS products isolated from cyanobacteria to date belong to the alkylresorcinol group (Martins et al. 2019). However, the close relationship of Clade 3.1 cyanobacterial enzymes with PKS18 from *M. tuberculosis* suggests that pyrone production by cyanobacteria could be possible. The function of cytochrome b5 in the biosynthesis of Clade 3.1 polyketides is yet to be verified, however, as mentioned previously, it may play a role in the synthesis of myolic acid, using the products of the type III PKS (Kolattukudy et al. 1997; Saxena et al. 2003).

Clade 3.2 was divided into Clades 3.21 and 3.22. Clade 3.21 comprised eukaryotic type III PKSs, including fungal and plant sequences, which partitioned further into distinct monophyletic groups. This topology suggests that type III PKSs in Eukaryota evolved from a common ancestor before the differentiation of the superkingdom, which is supported by previous studies (Mallika et al. 2011; Shimizu et al. 2017; Navarro-Muñoz and Collemare 2020). The fungal enzymes in Clade 3.21 are known to synthesize a variety of related compounds, including tri- and tetraketide pyrones (e.g., ORAS, CsyA), tetra- and pentaketide resorcinols (e.g., ORAS), and (alkenyl)-α pyrone (e.g., CsyB), using either malonyl- or ethylmalonyl-CoAs as extender units, and cyclizing the ketide with either lactonization or C2–C7 aldol condensation (Seshime et al. 2005; Funa et al. 2007; Seshime, Juvvadi, Kitamoto, Ebizuka, and Fujii 2010; Seshime, Juvvadi, Kitamoto, Ebizuka, Nonaka, et al. 2010; Hashimoto, Ishida, et al. 2013; Hashimoto, Seshime, et al. 2013). Most of the plant enzymes on the other hand (e.g., OLS, BAS, STS, CHS), synthesize flavonoid precursors from p-courmaroyl-CoA and use a variety of cyclization mechanisms (Flores-Sanchez and Verpoorte 2009), with a few showing a different substrate specificity and product formation (Eckermann et al. 1998; Mizuuchi et al. 2009) (supplementary table 3, Supplementary Material online).

Subclade 3.22 was comprised primarily of cyanobacterial type III PKSs (49 in total), including the hierridin biosynthesis enzyme, HidC (alkylresorcinol synthase). It was further divided into Clades 3.221 and 3.222. Clade 3.221 surprisingly included two *Azotobacter* type III PKSs (ArsB and ArsC) as well as unknown enzymes from cyanobacteria. It is speculated that ArsB and ArsC use ACP-bound long aliphatic thioesters rather than CoA-activated substrates in vivo, although they can use both substrates in vitro (Funa et al. 2006). Intramolecular cyclization occurs via C3–C8 aldol condensation and lactonization, respectively (supplementary table 3, Supplementary Material online). As the branches in Clade 3.221 are long, suggesting significant evolutionary distance, it is difficult to predict whether the cyanobacterial enzymes share a similar reaction mechanism to ArsB or ArsC.

Clade 3.222 was comprised exclusively of cyanobacterial sequences from hierridin-like biosynthesis gene clusters, which partitioned further into clades, 3.2221 and 3.2222. The gene clusters associated with Clade 3.2221 enzymes have a different genetic architecture to those associated with Clade 3.2222 enzymes, which closely resemble the *hidABC* gene cluster from *Cyanobium* sp. LEGE06113 (Costa et al. 2019) (fig. 2). Several of the subclade 3.2221 gene clusters encode two methyltransferases (HidB homologs), suggesting that the metabolites produced undergo an extra methylation step compared with the products of Clade 3.2222 pathways. Interestingly, the gene cluster associated with the type III PKS from *R. mediterranea* Skuja FSS1-150/1 (Clade 3.2221) also encodes a prenyltransferase (fig. 2), therefore this gene cluster could produce a completely novel compound. Prenyltransferases were not identified in any of the other gene clusters linked to this clade, suggesting that it was acquired through horizontal gene transfer. The only characterized enzyme in Clade 3.2222, HidC, putatively uses a C_14_ acyl-ACP thioester as a starter substrate, extending it with malonyl-CoA before cyclization through a C2-C7 aldol condensation (supplementary table 3, Supplementary Material online) (Costa et al. 2019). HidC-like PKS genes seem to be more abundant in marine cyanobacteria like *Prochlorococcus* and *Synechocystis*, whereas those encoding (7.7)paracyclophane synthases (Clade 2.222) are more prevalent in terrestrial species (supplementary table 2, Supplementary Material online). This suggests that the two different pathways could have evolved to facilitate ecological niche colonization by the producing organisms. However, our results also show that some terrestrial species, including *Nostoc* contain derivatives of the *hid* cluster. This could be due to horizontal gene transfer from one ecotype to another, as has been shown between type I PKS gene clusters in *Streptomyces* (Vicente et al. 2018).

In summary, our phylogenetic analysis suggests that modern cyanobacterial type III PKSs comprise three major lineages, including 1) enzymes associated with (7.7)-paracyclophane-like biosynthesis gene clusters (Clade 2.222), 2) enzymes associated with hierridin-like biosynthesis gene clusters (Clade 3.222), and 3) enzymes associated with cytochrome b5-encoding biosynthesis gene clusters (Clade 3.1) (fig. 3). All characterized cyanobacterial type III PKSs synthesize alkylresorcinols from long aliphatic acids (C_12_–C_16_) via a C2–C7 aldol cyclization mechanism, and it is likely that most of the uncharacterized enzymes also use this reaction mechanism, except for those in Clade 3.1, which probably use lactonization like their nearest homologues. The gross topology of the tree suggests that several different forces have shaped the evolution of type III PKSs in cyanobacteria, including vertical and horizontal gene transfer as well as duplication and gene loss events (this is also evident in the architecture of the corresponding gene clusters, fig. 2). For example, the proximity of cyanobacterial HidC (hierridin biosynthesis)-like enzymes (Clade 3.22) to the eukaryotic enzymes (Clade 3.21), suggests that an ancestral type III PKS was transferred from cyanobacteria to the progenitor of eukaryotes and its structure was conserved. Symbiogenesis, the evolution of eukaryotic organelles (e.g., chloroplasts) from prokaryotic organisms (e.g., cyanobacteria), lends further support to this theory (Cavalier-Smith 2002). Interestingly, nearly all plant type III PKSs investigated thus far are encoded within the nuclear genome not the chloroplast. However, it is possible that like many other genes originating from cyanobacteria, type III PKSs in plants were transferred to the nucleus during endosymbiosis (Timmis et al. 2004).

Gene duplication likely gave rise to multiple type III PKSs within single cyanobacterial species, as observed in *Microcystis* and *Nostoc* genomes. For example, in *Nostoc* sp. 335 mg, two hierridin-like biosynthesis gene clusters have evolved; most likely due to a duplication, followed by a methyltransferase duplication and introduction of an oxygenase (fig. 2). However, in most cases where two type III PKS clusters are present within a single organism, they are phylogenetically distinct, suggestive of horizontal gene transfer between phyla (fig. 3) (Zhaxybayeva et al. 2006). Horizontal transfer of genes between cyanobacteria and distantly related bacterial phyla could also explain the close phylogenetic relationship between cyanobacterial type III PKSs from Clade 3.21 and PKS18 from *Mycobacterium tuberculosis*, as well as cyanobacterial type III PKSs from Clade 3.22 and ArsC from *Azotobacter vinelandii.* Similarly, this might also explain the presence of lone cyanobacterial sequences in certain clades (e.g., *Moorea producens* PAL-8-15-08-1 in Clade 1 and *Mastigocoleus testarum* BC008 in Clade 2.1).

Presently, only two families of type III PKS metabolites have been characterized from cyanobacteria: the hierridins and the (7.7)paracyclophanes. However, our phylogenetic tree shows that at least three families are present, suggesting the potential repertoire of type III PKS products is more diverse than originally surmised. Characterized cyanobacterial type III PKSs appear to use very specific substrates in vivo (Bui et al. 2007; Nakamura et al. 2012; Preisitsch, Heiden, et al. 2016; Costa et al. 2019). This is comparable to bacterial and plant type III PKSs that show a rather rigid substrate specificity in vivo (Ghimire et al. 2008; Ghosh et al. 2008; Nakano et al. 2009; Nualkaew et al. 2012; Meslet-Cladiere et al. 2013). However, in vitro studies on purified bacterial type III PKSs suggest that they actually have a flexible substrate specificity (Zheng et al. 2001; Funabashi et al. 2008; Chemler et al. 2012; Hashimoto et al. 2014). Future in vitro studies might similarly reveal a broader substrate repertoire for cyanobacterial enzymes.

### Protein Modeling Highlights the Structural Diversity of Cyanobacterial Type III PKSs

The structure of type III PKS active sites is believed to provide clues to their substrate specificity and catalytic mechanism (Ferrer et al. 1999; Jez et al. 2001). Therefore, we modeled and compared the structures of cyanobacterial type III PKSs (*n* = 7) to previously elucidated crystal structures of bacterial and plant enzymes (*n* = 3) (supplementary table 4, Supplementary Material online) from our phylogenetic analysis (fig. 3). The size and topology of the active site cavities and their constitutive residues were closely examined in each case.

**Fig. 4.**
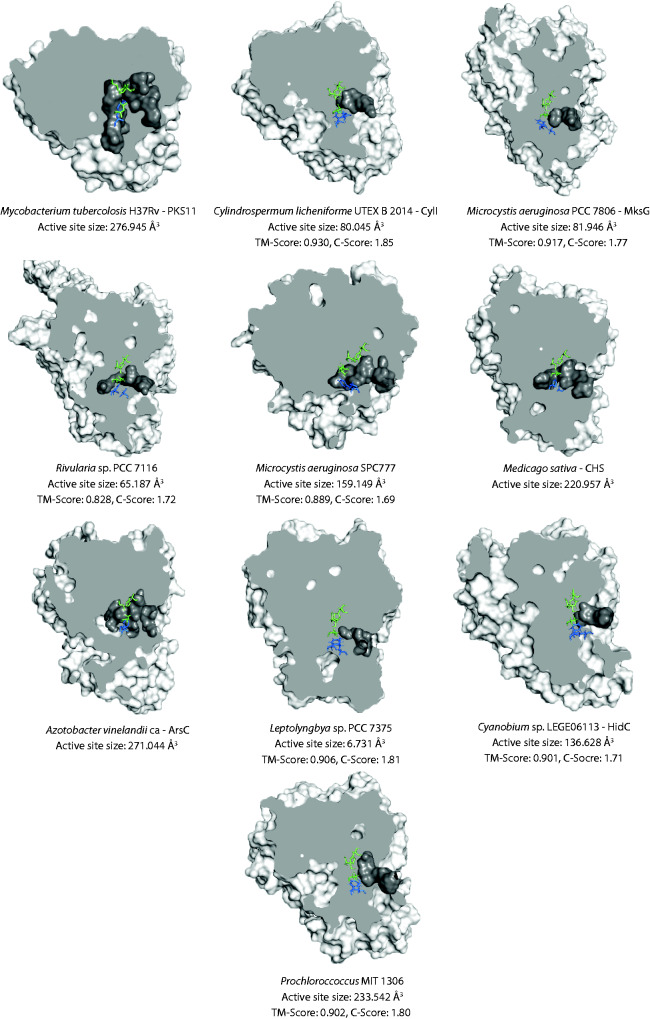
Protein models of representative type III PKSs from cyanobacteria, other bacteria, plants, and fungi. Structures were obtained through I-TASSER and visualized using PyMol2. A cross-section of the type III PKSs is displayed, with the active site cavity (dark gray) highlighted. Green residues are the conserved active site residues, Cys–His–Asn. Blue residues correspond to the so called “Gatekeeper” residues identified in CHS from *Medicago sativa*. The size of the active site cavity is given below the protein model. The crystal structures used to infer the models are listed in supplementary table 4, Supplementary Material online.

The catalytic triad of active site residues, Cys–Asn–His (green residues in fig. 4), as well as the overall topology, were conserved across all the cyanobacterial type III PKSs modeled when compared with known crystal structures. Interestingly, the hydrophobic nonpolar “Gatekeeper” residues identified in plant CHS, Phe215, and 265 (numbering from CHS from *Medicago sativa*) (blue residues in fig. 4), were only partially conserved in the bacterial sequences. Residues corresponding to Phe265 in CHS were particularly heterogenous across the enzymes examined, and phylogenetic correlations were observed (supplementary fig. 5, Supplementary Material online). Although Clade 3.2222 enzymes, including HidC, which is predicted to use acyl-ACP thioesters as substrates to form alkylresorcinols (Costa et al. 2019), had a conserved methionine (hydrophobic nonpolar) substitution at this position, all Clade 3.1 enzymes had a cysteine (sulfur-containing polar) residue. The substrate and product for this group are still unknown. Finally, Clade 2.222 enzymes, which use C_12_ thioesters obtained from a type I PKS to produce alkylresorcinols, which are further dimerized into (7.7)paracyclophanes, had either a glutamine or an asparagine (polar uncharged) residue at this position. A possible explanation for these confounding results is that CylI, and closely related characterized Clade 2 cyanobacterial enzymes, have evolved a different mechanism to tether and process their substrates. For instance, the characterized cyanobacterial type III PKSs from clade 2.222 receive their substrates from the ACP-module rather than taking up the substrate directly from a CoA or from a single ACP unit (Nakamura et al. 2012).

Next, the size of the active sites was investigated. The size for each enzyme was predicted using the online software CASTp. The size of the active sites analyzed ranged between 6.731 Å^3^ (*Leptolyngbya* sp. PCC 7375) and 276.945 Å^3^ (PKS11 from *M. tuberculosis* H37Rv), with a mean of 153.217 Å^3^ (fig. 4). The cyanobacterial active sites were generally smaller (mean = 109.035 Å^3^) than other bacterial (mean = 261.083 Å^3^) and CHS from plants (220.957 Å^3^) cavities. Previous studies have demonstrated a link between the size of the active site cavity and the size of the substrates handled by the type III PKS (Jez et al. 2000).

As most of the cyanobacterial enzymes are predicted to use long aliphatic acid substrates, it was surprising that many had relatively small active site cavities, compared with their bacterial counterparts, regardless of their phylogeny (figs. 3 and 4). Notably, the active sites of Clade 2 enzymes from *C. licheniforme* UTEX B 2014 (CylI, 80.045 Å^3^) and *M. aeruginosa* PCC 7806 (MksG, 81.946 Å^3^) are predicted to be approximately half the size of the average type III PKS enzyme cavity. We expected CylI (and its homologues) to have a large active site like that of PKS11 from *M. tuberculosis* H37Rv (Clade 2; 276.945 Å^3^), which uses similar substrates. Similarly, ArsC from *A. vinelandii* has an active site of 271.044 Å^3^ whereas HidC is predicted to be 136.628 Å^3^; only half the size (fig. 4). The only difference between these cyanobacterial and non-cyanobacterial type III PKSs is the cyclization event; where ArsC and PKS11 make pyrones, HidC and CylI cyclize their products into resorcinols. This might indicate that the larger active site is needed for the formation of the pyrone structure, whereas the smaller active site might be better suited to the production of resorcinols. This has also been shown in trORAS, where a mutation in Phe252 to glycine disrupted resorcinol production and increased pyrone production (Rubin-Pitel et al. 2008). As glycine is smaller than phenylalanine, this mutation is bound to increase the size of the active site. Exactly how the cyclization occurs is still unclear, likewise, it is unknown if this happens inside the enzyme or as a spontaneous event after the release of the ketide chain from the enzyme (Austin, Bowman, et al. 2004; Li et al. 2011; Wu et al. 2012; Satou et al. 2013). These results suggest that cyclization occurs while the ketide chain is within the active site. However, further research is needed to verify this.

Most of the predicted active site cavities of the cyanobacterial enzymes from Clade 3 were larger than those from Clade 2, except for the *Leptolyngbya* (6.731 Å^3^) and *Rivularia* sp. PCC 7116 (65.187 Å^3^) enzymes. However, they were still generally smaller than their noncyanobacterial counterparts. The extremely small size of the *Leptolyngbya* sp. PCC 7375 enzyme cavity suggests that it might not be functional or produces a very small polyketide, as the size of the active site of PKSs is generally proportional to the number of cyclization events (Austin, Izumikawa, et al. 2004; Rubin-Pitel et al. 2008; Wu et al. 2012). The small active site of the *Rivularia* sp. PCC 7116 enzyme suggests that it produces resorcinols rather than pyrones, despite its phylogenetic proximity to the pyrone synthase (PKS18) from *M. tuberculosis* H37Rv. On the other hand, the type III PKS from *M. aeruginosa* SPC777 has a relatively large active site cavity, which might be able to sustain pyrone formation (Sankaranarayanan et al. 2004). Likewise, the putative type III PKS from *Prochlorococcus* MIT 1306 is predicted to have a large active site (233.542 Å^3^) and, much like plant and bacterial type III PKSs, could potentially sustain pyrone formation. Hence, pyrone formation by cyanobacterial type III PKSs should not be excluded even though to date, no pyrones have been isolated from cyanobacteria (Martins et al. 2019). In summary, protein modeling highlights the significant structural diversity present among cyanobacterial type III PKSs however, further research is needed to confirm if this structural diversity translates to functional diversity.

## Conclusions

This study demonstrated that gene clusters encoding type III PKS are present in 16.8% of sequenced cyanobacterial genomes. Comparison of type III PKS biosynthesis gene cluster architecture and phylogenetic analysis showed that type III PKSs in cyanobacteria evolved into three major lineages, including enzymes associated with 1) (7.7)paracyclophane-like biosynthesis gene clusters, 2) hierridin-like biosynthesis gene clusters, and 3) cytochrome b5 genes. The evolutionary history of these enzymes is complex, with some sequences partitioning primarily according to speciation and others putatively according to their reaction type. Protein modeling indicated that cyanobacterial type III PKSs have a similar overall topology to plant and bacterial enzymes, although their active site cavities are usually smaller. Our results also showed that the size of the active site cavity does not necessarily correspond to the size of the substrate used by type III PKSs or the number of extensions catalyzed. Although the catalytic triad of amino acids (Cys–Asn–His) is conserved in cyanobacteria, one of the so-called “Gatekeeper” amino acid residues (Phe265 in CHS) is not, suggesting that it might not be important for substrate specificity in cyanobacterial type III PKSs. Overall, our protein modeling highlights the structural diversity of type III PKSs from different taxa.

As only four cyanobacterial type III PKS gene clusters have been experimentally characterized, the repertoire of compounds is largely unexplored, and future studies are needed to identify the prevalence of different type III PKS metabolites from cyanobacteria, and their biological function. As the previously identified products have diverse bioactivities (antiplasmodial, cytotoxic, UV blocking), cyanobacteria remain a potentially rich, yet untapped source of biomedically relevant type III polyketides, including potentially prenylated, hydroxylated, and sulfonated type III PKSs and their novel biosynthetic enzymes.

## Supplementary Material

Supplementary data are available at *Genome Biology and Evolution* online.

## Supplementary Material

evab056_Supplementary_Data
