# Supplementary material for: Genome Mining and Evolutionary Analysis Reveal Diverse Type III Polyketide Synthase Pathways in Cyanobacteria
Source: Genome Biol Evol. 2021 Mar 19;13(4):evab056. doi: 10.1093/gbe/evab056 (PMC8086630; doi:10.1093/gbe/evab056)
Supplement: evab056_Supplementary_Data [file evab056_supplementary_data.pdf]

Supplementary Table 1. List of type III PKSs and genomes analysed in this study

Name	Organism	GenBank Accession	Genome Accession	Reference
OKS2	*Aloe arborescens*	ACR19997		(Mizuuchi, et al. 2009)
DpgA	*Amycolatopsis balhimycina* DSM 5908	CAC48378		(Pfeifer, et al. 2001)
DpgA	*Amycolatopsis orientalis* NRRL 18098	G4V4T4		(Chen, et al. 2001)
	Aphanothece cf. minutissima CCALA 015		ASM300392v1	
LAP5	*Arabidopsis thaliana*	O23674		(Mizuuchi, et al. 2008)
LAP6	*Arabidopss thaliana*	Q8LDM2		(Mizuuchi, et al. 2008)
CsyA	*Aspergillus oryzae* RIB40	BAD97390		(Seshime, et al. 2010)
CsyB	*Aspergillus oryzae* RIB40	BAD97391		(Hashimoto, et al. 2013)
ArsB	*Azotobacter vinelandii* ca	ZP_00418325		(Funa, et al. 2006)
ArsC	*Azotobacter vinelandii* ca	ZP_00418326		(Funa, et al. 2006)
BcsB	*Bacillus* cs100	MW218666		This study
BcsA	*Bacillus subtilis* 168	CEI57418		(Nakano, et al. 2009)
	Calothrix sp. HK-06		ASM190474v1	
	Calothrix sp. NIES-4071		ASM236845v1	
	Calothrix sp. NIES-4105		ASM236841v1	
CHS3	*Camellia sinensis*	P48388		(Takeuchi, et al. 1994)
OLS	*Cannabis sativa*	BAG14339		(Taura, et al. 2009)
	Chamaesiphon minutus PCC 6605		ASM31714v1	
	Chamaesiphon polymorphus CCALA 037		ASM300384v1	
	Chlorogloeopsis fritschii PCC 6912		ChlPCC6912_1	
	Chlorogloeopsis fritschii PCC 9212		ChlPCC9212_1	
CHS1	*Citrus sinensis*	Q9XJ58		(Moriguchi, et al. 1999)
	Cyanobium gracile PCC 6307		ASM31651v1	
HidC	*Cyanobium* sp. LEGE06113	QBC65480		(Costa, et al. 2019)
	Cyanobium sp. NIES-981		ASM90008853v 1	
	Cyanobium sp. PCC 7001		ASM15563v1	
	Cyanobium usitatum str. Tous		ASM301188v1	
	Cyanothece sp. PCC 7424		ASM2182v1	
CylI	*Cylindrospermum lichenforme* UTEX B 2014	AFV96143		(Nakamura, et al. 2012)
	Cylindrospermum stagnale PCC 7417		ASM31753v1	
FabH	*Escherichia coli* (Outgroup)	WP_000288132		
	Filamentous cyanobacterium CCP2		ASM300361v1	
BPS	*Garcinia mangostena*	L7NCQ3		(Nualkaew, et al. 2012)
	Gloeocapsa sp. PCC 73106		ASM33203v1	
	Gloeocapsa sp. PCC 7428		ASM31755v1	
	Gloeocapsopsis sp. AAB1		ASM296486v1	
CHS	*Hydrangea macrophylla*	BAA32732		(Akiyama, et al. 1999)
	Leptolyngbya sp. NIES-2104		ASM148521v1	
	Leptolyngbya sp. PCC 7375		ASM31611v1	
	Leptolyngbya valderiana BDU 20041		ASM163739v1	
	Mastigocoleus testarum BC008		ASM145602v1	
CHS	*Medicago sativa*	P30075		(McKhann and Hirsch 1994)
CHS2	*Medicago sativa*	P30074		(Jez, et al. 2000)
	Microcystis aeruginosa CHAOHU 1326		ASM189532v1	
	Microcystis aeruginosa DIANCHI905		MicAerD1.0	
	Microcystis aeruginosa NIES-1211		ASM320662v1	
	Microcystis aeruginosa NIES-2481		ASM170495v2	
	Microcystis aeruginosa NIES-2549		ASM98178v2	
	Microcystis aeruginosa NIES-298		ASM289727v1	
	Microcystis aeruginosa NIES-298		ASM289731v1	
	Microcystis aeruginosa NIES-87		ASM293383v1	
	Microcystis aeruginosa PCC 7005		Mic70051.0	
MksG	*Microcystis aeruginosa* PCC 7806	AM778955		(Frangeul, et al. 2008)
	Microcystis aeruginosa PCC 7806SL		ASM209597v1	
	Microcystis aeruginosa PCC 7941		ASM31220v1	
	Microcystis aeruginosa PCC 9432		ASM30799v2	

	Microcystis aeruginosa PCC 9701		ASM31228v1	
	Microcystis aeruginosa PCC 9809		ASM31226v1	
	Microcystis aeruginosa Sj		ASM320655v1	
	Microcystis aeruginosa SPC777		spc777-v1	
	Microcystis aeruginosa TAIHU98		MicAerT1.0	
	Microcystis sp. MC19		ASM301973v1	
	Moorea bouillonii PNG NPG5-198		ASM194249v1	
	Moorea producens JHB		ASM185420v1	
	Moorea producens PAL-8-15-08-1		ASM176723v1	
PKS10	*Mycobacterium marinum* M	CDM76540		(Parvez, et al. 2018)
PKS11	*Mycobacterium marinum* M	CDM76544		(Parvez, et al. 2018)
PKS10	*Mycobacterium tuberculosis* H37Rv	P9WPF5		(Sirakova, et al. 2003)
PKS11	*Mycobacterium tuberculosis* H37Rv	P9WPF1		(Saxena, et al. 2003)
PKS18	*Mycobacterium tuberculosis* H37Rv	P9WPF3		(Saxena, et al. 2003)
ORAS	*Neurospora crassa* OR74A	EAA31191		(Funa, et al. 2007)
	Nostoc sp. 3335mG		ASM318586v1	
CabI	*Nostoc* sp. CAVN2	AMB48450		(Preisitsch, et al. 2016)
	Nostoc sp. NIES-4103		ASM236833v1	
MerE	*Nostoc* sp. UIC10110	AQA28564		(May, et al. 2017)
	Oscillatoriales cyanobacterium MTP1		ASM148274v2	
STS	*Pinus sylvestris*	Q02323		(Schanz, et al. 1992)
	Pleurocapsa sp. PCC 7319		ASM33219v1	
	Prochlorococcus marinus MIT 1342		ASM163214v1	
	Prochlorococcus marinus MIT9313		ASM1148v1	
	Prochlorococcus marinus str. MIT 1320		ASM163207v1	
	Prochlorococcus marinus str. MIT 1323		ASM163202v1	
	Prochlorococcus sp. MIT 0701		ASM76029v1	
	Prochlorococcus sp. MIT 0702		ASM76031v1	
	Prochlorococcus sp. MIT 0703		ASM76033v1	
	Prochlorococcus sp. MIT 1303		ASM163196v1	
	Prochlorococcus sp. MIT 1306		ASM163198v1	
	Pseudanabaena biceps PCC 7429		ASM33221v1	
	Pseudanabaena sp. 'Roaring Creek'		ASM140279v1	
	Pseudanabaena sp. BC1403		ASM291458v1	
	Pseudanabaena sp. SR411		ASM225194v1	
PhlD	*Pseudomonas fluorescens* Q2-87	ADG03656		(Bangera and Thomashow 1999)
	Raphidiopsis mediterranea	MW218665		This study
BAS	*Rheum plamatum*	AAK82824		(Abe, et al. 2001)
	Rivularia sp. PCC 7116		ASM31666v1	
	Rubidibacter lacunae KORDI 51-2		KS51_v1	
PKS1	*Rubus ideaus*	ACF72868		(Zheng, et al. 2001)
PKS3	*Rubus ideaus*	AAK15176		(Zheng, et al. 2001)
PKS4	*Rubus ideaus*	B0LDU5		(Zheng, et al. 2001)
PKS5	*Rubus ideaus*	ABV54603		(Zheng, et al. 2001)
RPPA	*Saccharopolyspora erythraea* E_8-7	AAL78053		(Cortés, et al. 2002)
RPPA	*Streptomcyes griseus* NBRC 13350	BAB91443		(Funa, et al. 2002)
RPPB	*Streptomcyes griseus* NBRC 13350	BAB91444		(Funa, et al. 2002)
SrsA	*Streptomcyes griseus* NBRC 13350	BAG17301		(Funabashi, et al. 2008)
Gcs	*Streptomyces coelicolor* A3(2)	NP_631277		(Gruschow, et al. 2007)
THNS	*Streptomyces coelicolor* A3(2)	Q9FCA7		(Izumikawa, et al. 2003)
RPPA	*Streptomyces griseus* IFO 133350	Q54240		(Ueda, et al. 1995)
RPPA	*Streptomyces lividans* TK21	BAB91445		(Funa, et al. 2002)
TotC1	*Streptomyces pactum* SCSIO 02999	ATL73040		(Chen, et al. 2017)
RPPA	*Streptomyces peucetius* ATCC 27952	B0FYK7		(Ghimire, et al. 2008)
StTs	*Streptomyces toxytricini* NRRL 15443	AEO44526		(Zeng, et al. 2012)
DpgA	*Streptomyces toyocaensis* NRRL 15009	AAM80548		(Pootoolal, et al. 2002)
VemA	*Streptomyces venezuelae* ATCC 10712	WP_150158909		(Thanapipatsiri, et al. 2016)
Ken2	*Streptomyces violaceoruber* DSM41773	CAQ52620		(Wenzel, et al. 2008)
	Synechococcus sp. 1G10		ASM225262v1	
	Synechococcus sp. 8F6		ASM225266v1	
	Synechococcus sp. BL107		ASM15380v1	

	Synechococcus sp. CC9311		ASM1458v1	
	Synechococcus sp. CC9616		ASM51523v1	
	Synechococcus sp. CC9902		ASM1250v1	
	Synechococcus sp. KORDI-100		ASM73753v1	
	Synechococcus sp. LL		ASM225270v1	
	Synechococcus sp. MIT S9504		ASM163210v1	
	Synechococcus sp. MIT S9509		ASM163193v1	
	Synechococcus sp. MW101C3		ASM225263v1	
	Synechococcus sp. P1 UW179A		UW179A	
	Synechococcus sp. P1 UW179B		UW179B	
	Synechococcus sp. RS9917		ASM15306v1	
	Synechococcus sp. SynAce01		ASM188521v1	
	Synechococcus sp. WH 5701		ASM15304v1	
	Synechococcus sp. WH 7805		ASM15328v1	
	Synechococcus sp. WH 8016		ASM23067v1	
	Synechococcus sp. WH 8020		ASM104084v1	
	Synechococcus sp. WH 8102		ASM19597v1	
	Synechococcus sp. WH 8103		WH8103.1	
	Synechococcus WH7803		ASM6350v1	
	Tolypothrix bouteillei VB521301		ASM76069v2	

Supplementary Table 2. Summary of cyanobacterial genome mining results for type III PKS biosynthesis gene clusters

Order	Genomes mined	Genomes with type III PKS	Relative abundance (%)	Cluster type				Type III PKS genes
				(i)	(ii)	(iii)	(iv)	
Chrooccales	63	22	34.92	14	1	8	0	23
Chroococcidiopsidales	5	0	0	0	0	0	0	0
Gloeobacterales	5	3	60	1	0	2	0	3
Gloeoemargaritales	1	0	0	0	0	0	0	0
Nostocales	171	12	7.02	6	3	3	1	13
Oscillatoriales	54	4	7.41	3	1	0	1	5
Pleurocapsales	8	1	12.5	1	0	0	0	1
Spirulinales	2	0	0	0	0	0	0	0
Synechococcales	197	44	22.34	0	43	2	0	45
Unknown	11	1	9.09	0	0	1	0	1
**TOTAL**	517	87	16.83	25	48	16	2	91

Genomes mined using antiSMASH 5.1 (Blin, et al. 2019). Gene cluster types; (i) (7.7)paracyclophane-like, (ii) hierridin-like, (iii) gene clusters encoding cytochrome b5, (iv) gene clusters that do not belong to any of the major cyanobacterial branches.

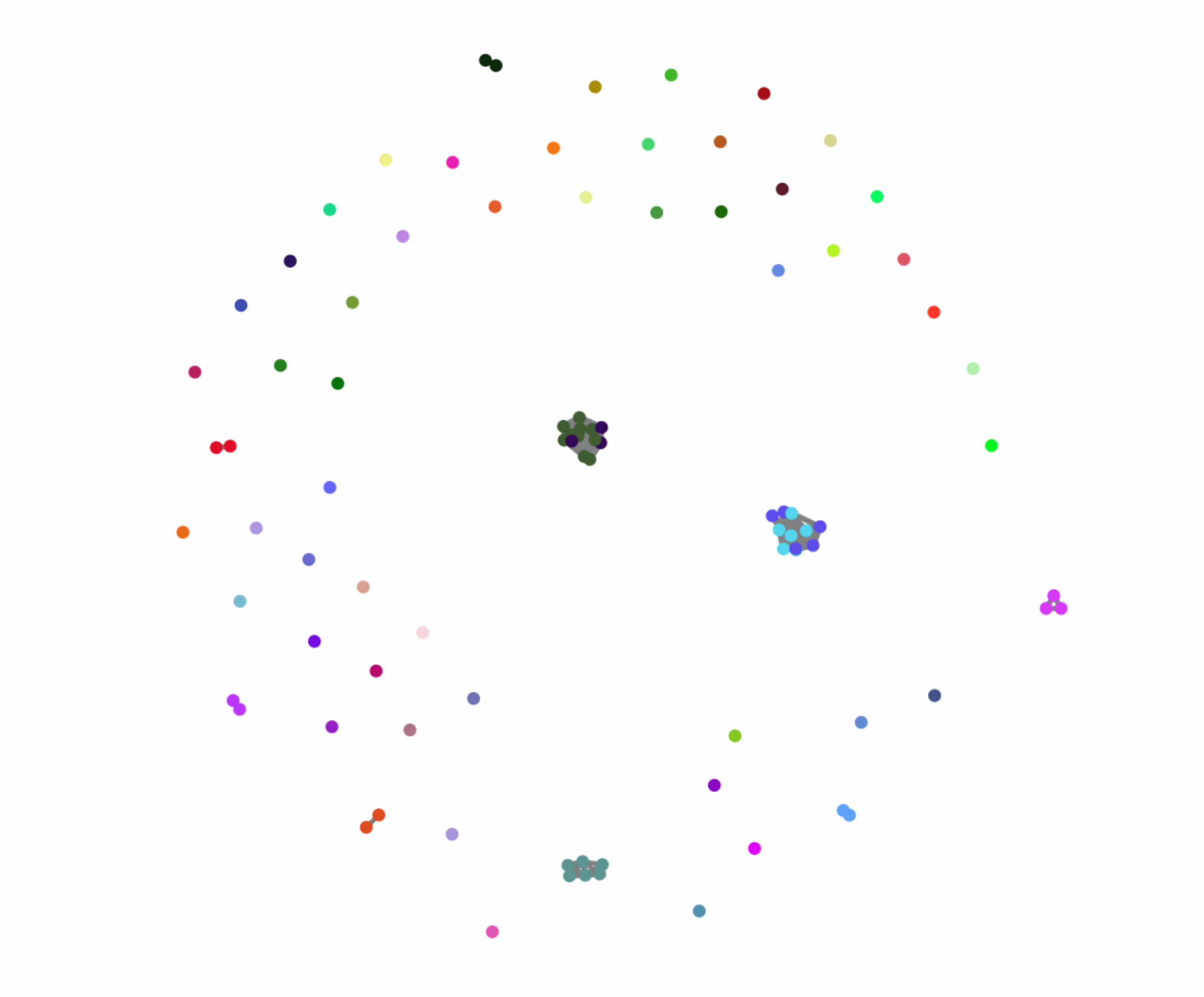

Supplementary Figure 1. Type III PKS gene cluster network. A visual representation of the gene cluster network associated with type III PKSs from the phylogenetic analysis (Figure 3) identified by BIG-SCAPE (Navarro-Munoz, et al. 2020). Each dot represents a gene cluster. Lines between two or more dots indicate homologous gene clusters. Based on the data mined from publicly available genomes, 60 families are present, where 49 of these are singletons (gene cluster families with only one member).

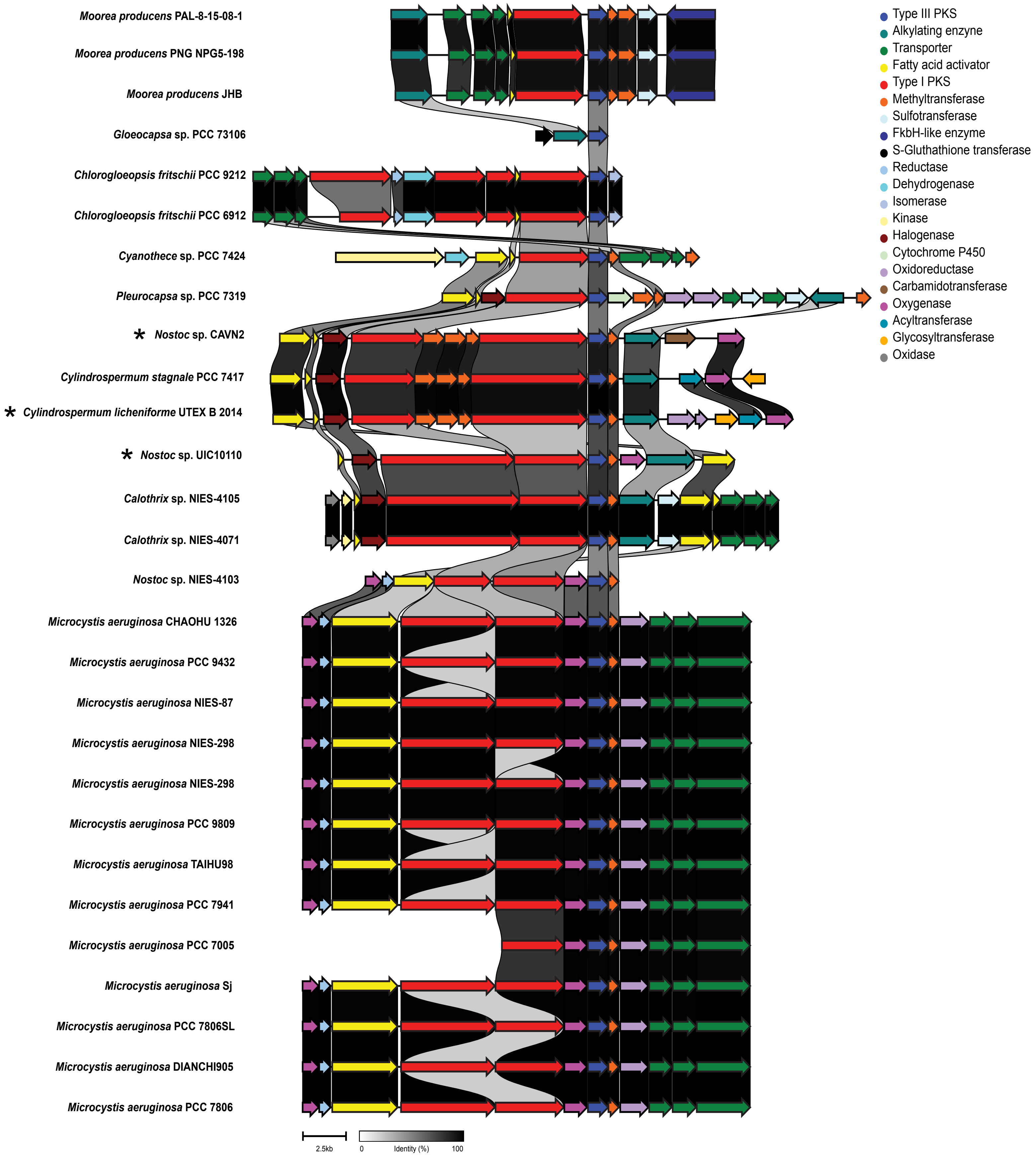

Supplementary Figure 2. Comparison of characterised and predicted (7.7)paracyclophane-like biosynthesis gene clusters. Gene clusters were compared using Clinker v 0.0.12 (Gilchrist and Chooi 2021). Open reading frames are colour-coded according to their predicted function. Gene clusters are arranged according to the position of their associated type III PKS in the phylogenetic tree (Clade 2.222, Figure 3).

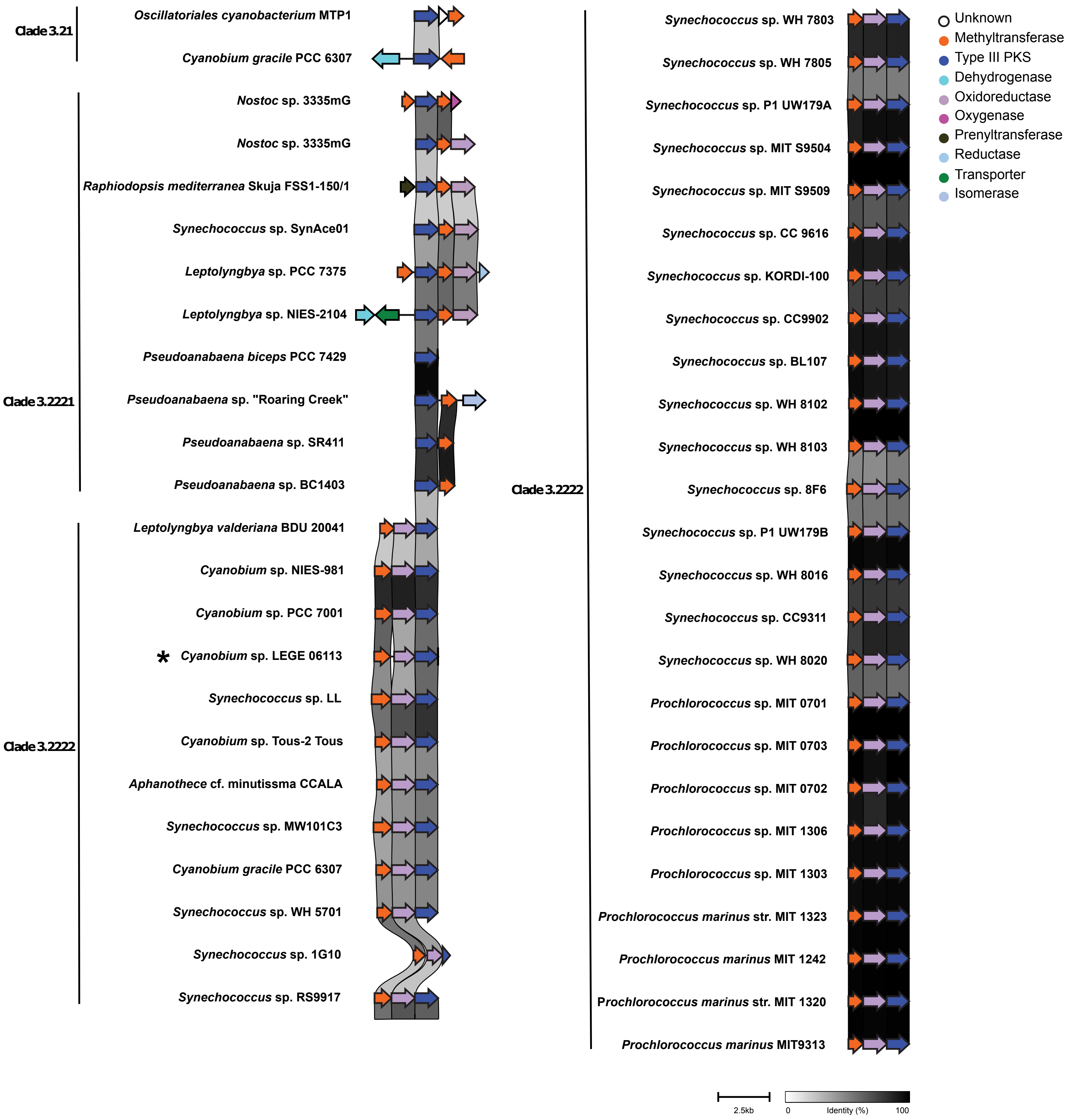

Supplementary Figure 3. Comparison of characterised and predicted hierridin-like biosynthesis gene clusters. Gene clusters were compared using Clinker v 0.0.12 (Gilchrist and Chooi 2021). Open reading frames are colour-coded according to their predicted function. Gene clusters are arranged according to the position of their associated type III PKS in the phylogenetic tree (Clades 3.221, 3.2221 and 3.2222, Figure 3). Known Hierridin producer has been indicated.

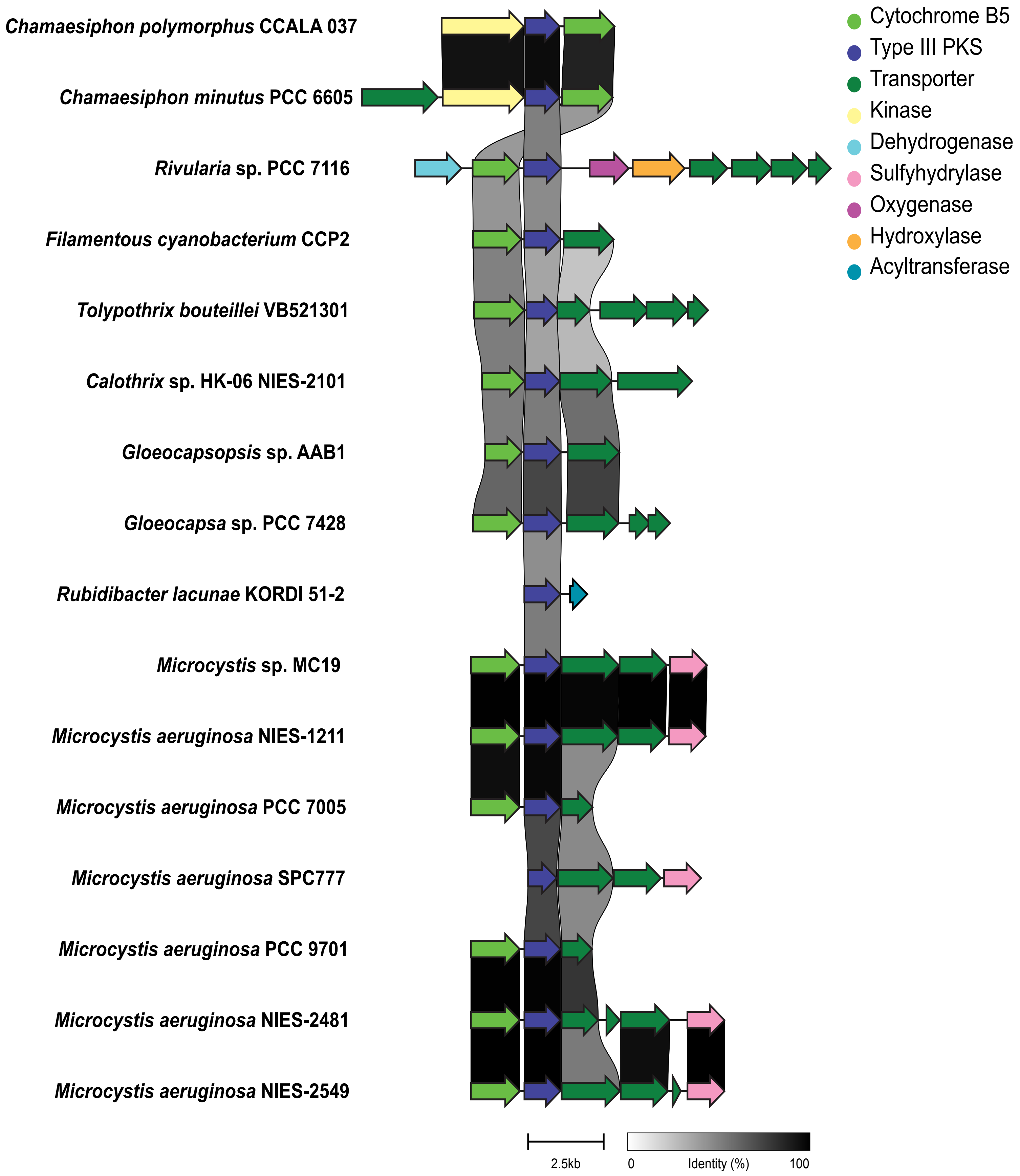

Supplementary Figure 4. Comparison of characterised and predicted type III PKS gene clusters associated with cytochrome b5. Gene clusters were compared using Clinker v 0.0.12 (Gilchrist and Chooi 2021). Open reading frames are colour-coded according to their predicted function. Gene clusters are arranged according to the position of their associated type III PKS in the phylogenetic tree (Clade 3.1, Figure 3).

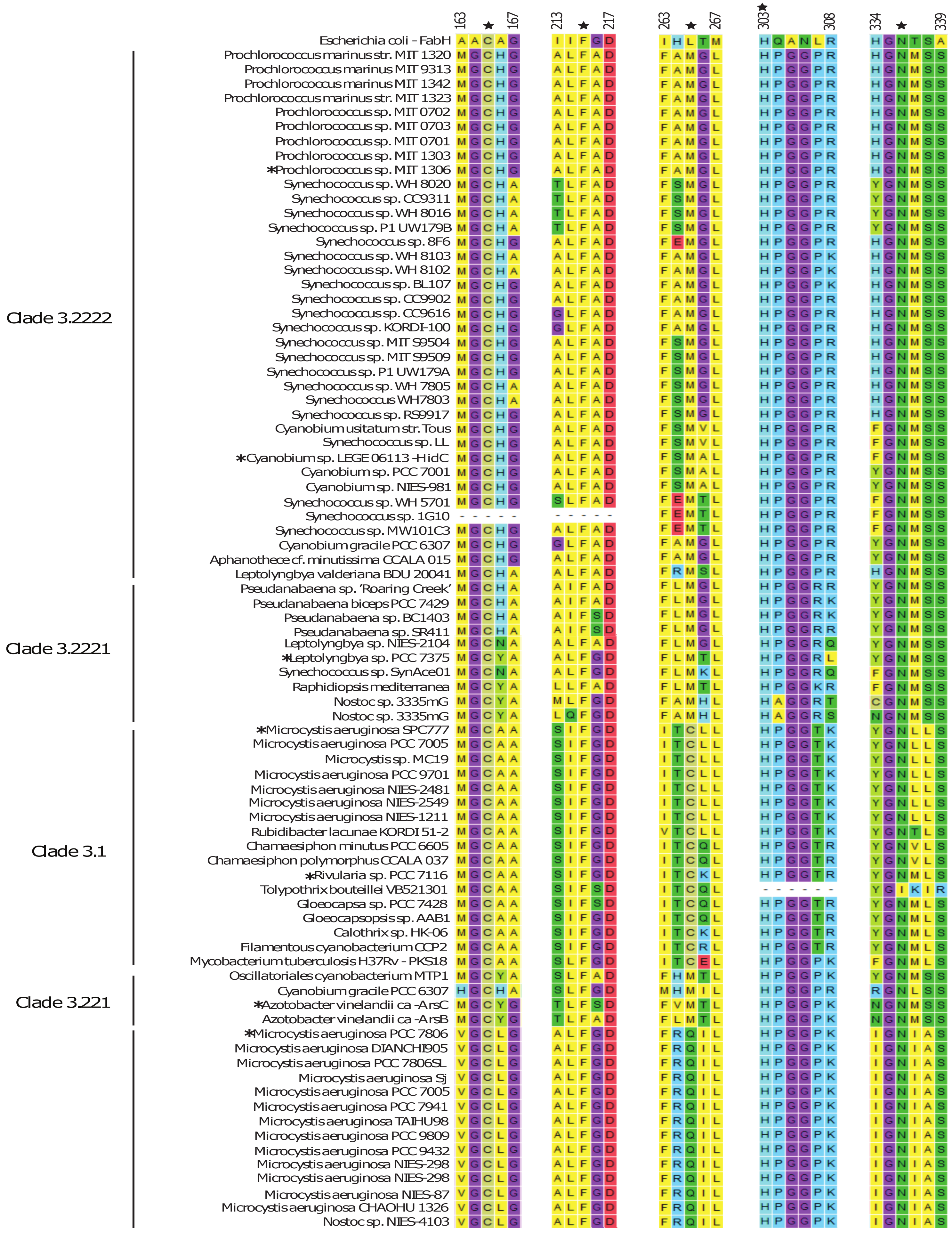

Continued next page

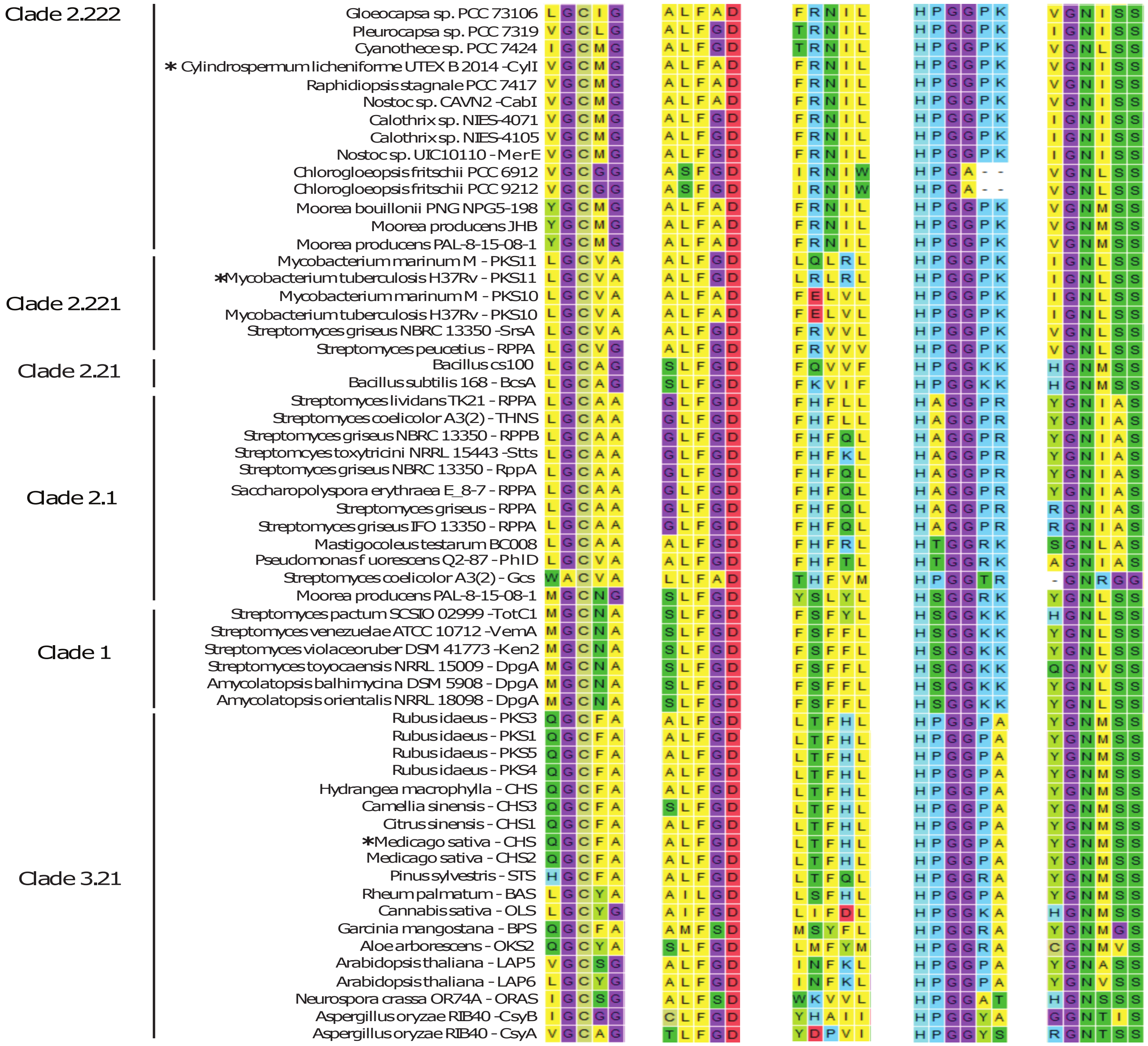

Supplementary Figure 5. Alignment of the type III PKS sequences used to create the phylogenetic tree (Figure 3). Alignment performed using MUSCLE through MEGA7 (Edgar 2004). Numbers above the alignment correspond to amino acid residues from CHS (*Medigaco sativa*), with catalytic active residues and 'Gatekeeper' residues marked with a star. Clades are annotated according to the corresponding phylogenetic tree (Figure 3). The asterisk denotes the sequences of enzymes that were also modelled (Figure 4).

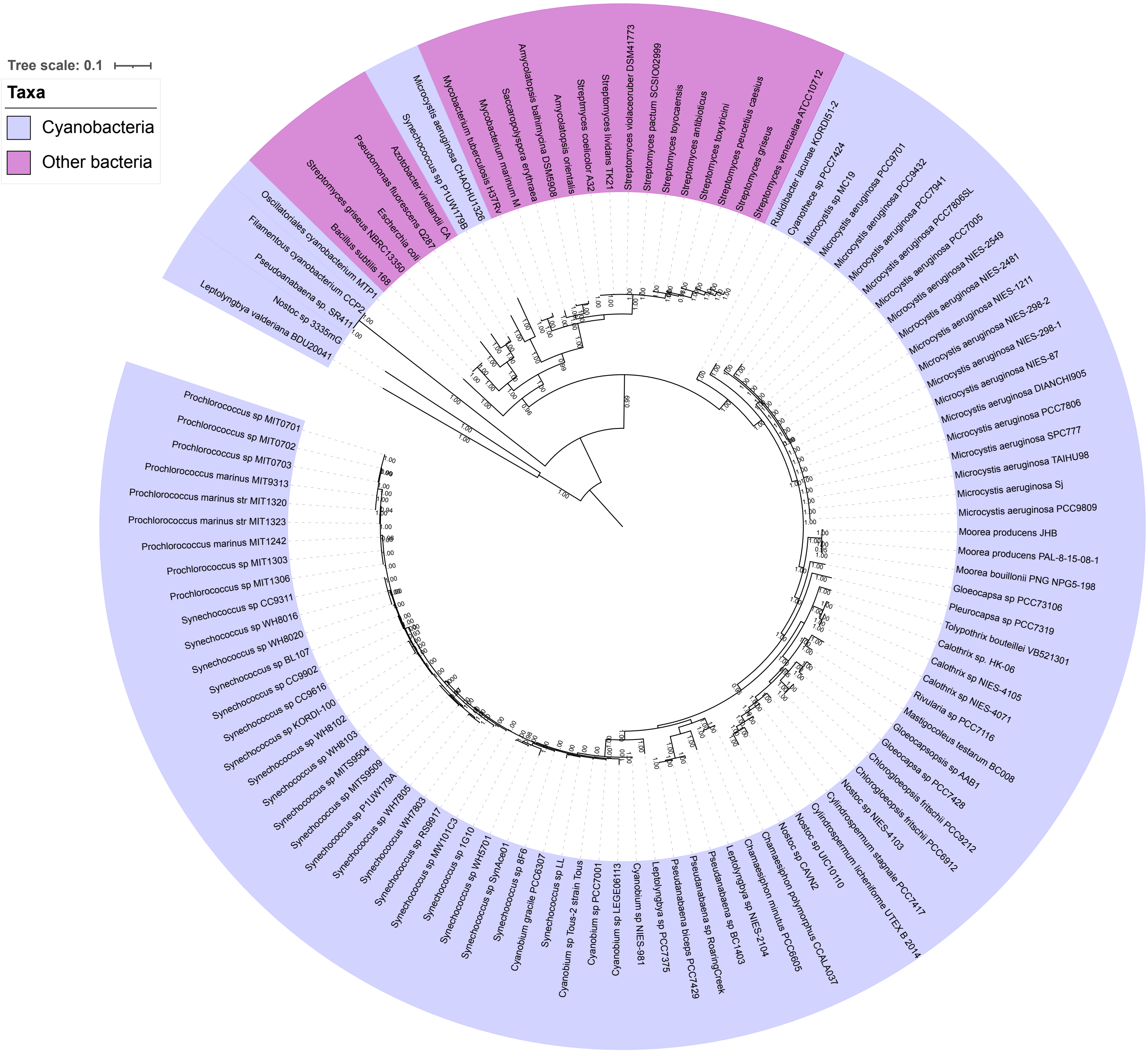

Supplementary Figure 6. Phylogenetic species (16S rRNA gene) tree for cyanobacteria and other bacteria investigated in this study. The tree was generated using MrBayes (Ronquist, et al. 2012) with the JC model. Colour-coding according to speciation; cyanobacteria (light blue), other bacteria (purple). Bootstrap values from 1,000,000 replicates above a threshold of 0.9 are shown on branches. The scale bar represents 0.1 nucleotide substitutions per nucleotide site.

Supplementary Table 3. Previously characterised type III PKSs used in this study and their reaction mechanisms

PKS III	Organism	Reaction Type	Major Product Group	Clade
		**CYANOBACTERIAL ENZYMES**		
**HidC**	*Cyanobium* sp. LEGE06113	C2-C7 Aldol	Alkylresorcicols	3.2222
**CylI**	*Cylindrospermum lichenforme* UTEX B 2014	C2-C7 Aldol	Alkylresorcicols	2.222
**CabI**	*Nostoc* sp. CAVN2	C2-C7 Aldol	Alkylresorcicols	2.222
**MerE**	*Nostoc* sp. UIC10110	C2-C7 Aldol	Alkylresorcicols	2.222
		**BACTERIAL ENZYMES**		
**DpgA**	*Amycolatopsis balhimycina* DSM 5908	C8-C3 Claisen	3.5-Dihydroxyphenylacetyl-CoA	1
**DpgA**	*Amycolatopsis orientalis* NRRL 18098	C8-C3 Claisen	3.5-Dihydroxyphenylacetyl-CoA	1
**ArsB**	*Azotobacter vinelandii* ca	C3-C8 Aldol	Alkylresorcinols	3.221
**ArsC**	*Azotobacter vinelandii* ca	Lactonization	Alkylpyrones	3.221
**BcsB**	*Bacillus* cs100	-	-	2.21
**BcsA**	*Bacillus subtilis* 168	Lacotnization	Alkylpyrones	2.21
**Pks10**	*Mycobacterium marinum* M	Lactonization	Methylated alkylpyrones	2.21
**Pks11**	*Mycobacterium marinum* M	Lactionization	Methylated alkylpyrones	2.21
**PKS10**	*Mycobacterium tuberculosis* H37Rv	Lactonization	Methylated alkylpyrones	2.21
**PKS11**	*Mycobacterium tuberculosis* H37Rv	Lactonization	Methylated alkylpyrones	2.21
**PKS18**	*Mycobacterium tuberculosis* H37Rv	Lactonization	Alkylpyrones	3.1
**PhID**	*Pseudomonas fluorescens* Q2-87	C6-C1 Claisen	Phloroglucinol	2.1
**RPPA**	*Saccharopolyspora erythraea* E_8-7	C3-C8 Aldol	Tetrahydroxynapthalene	2.1
**SrsA**	*Streptomcyes griseus* NBRC 13350	C6-C1 Claisen	Methylated resorcinols	2.21
**RPPA**	*Streptomcyes griseus* NBRC 13350	C3-C8 Aldol	Tetrahydroxynapthalene	2.1
**RPPB**	*Streptomcyes griseus* NBRC 13350	C3-C8 Aldol	Tetrahydroxynapthalene	2.1
**THNS**	*Streptomyces coelicolor* A3(2)	C2-C7 Aldol	Tetrahydroxynapthalene	2.1
**Gcs**	*Streptomyces coelicolor* A3(2)	Lactonization	Germicidins	2.1
**RPPA**	*Streptomyces griseus* IFO 133350	C3-C8 Aldol	Tetrahydroxynapthalene	2.1
**RPPA**	*Streptomyces lividans* TK21	C3-C8 Aldol	Tetrahydroxynapthalene	2.1
**TotC1**	*Streptomyces pactum* SCSIO 02999	C8-C3 Claisen	3.5-Dihydroxyphenylacetyl-CoA	1
**RPPA**	*Streptomyces peucetius* ATCC 27952	C3-C8 Aldol	Tetrahydroxynapthalene	2.21
**Stts**	*Streptomyces toxytricini* NRRL 15443	C3-C8 Aldol	Tetrahydroxynapthalene	2.1
**DpgA**	*Streptomyces toyocaensis* NRRL 15009	C8-C3 Claisen	3.5-Dihydroxyphenylacetyl-CoA	1
**VemA**	*Streptomyces venezuelae* ATCC 10712	C8-C3 Claisen	3.5-Dihydroxyphenylacetyl-CoA	1
**Ken2**	*Streptomyces violaceoruber* DSM41773	C8-C3 Claisen	3.5-Dihydroxyphenylacetyl-CoA	1
		**PLANT ENZYMES**		
**OKS2**	*Aloe arborescens*	C6-C11 Aldol	Barbaloin	3.21
**LAP5**	*Arabidopsis thaliana*	Lactonization	Alkylpyrones	3.21
**LAP6**	*Arabidopss thaliana*	Lactonization	Alkylpyrones	3.21
**CHS3**	*Camellia sinensis*	C6-C1 Claisen	Narigenin	3.21
**OLS**	*Cannabis sativa*	C2-C7 Aldol	Olivetol	3.21
**CHS1**	*Citrus sinensis*	C6-C1 Claisen	Narigenin	3.21
**BPS**	*Garcinia mangostena*	C6-C1 Claisen	Benzophenone	3.21
**CHS**	*Hydrangea macrophylla*	C6-C1 Claisen	Narigenin	3.21
**CHS**	*Medicago sativa*	C6-C1 Claisen	Narigenin	3.21
**CHS2**	*Medicago sativa*	C6-C1 Claisen	Narigenin	3.21
**STS**	*Pinus sylvestris*	C7-C2 Aldol	Stilbene	3.21
**BAS**	*Rheum plamatum*	Loss of C1	Benzalacetone	3.21
**PKS1**	*Rubus ideaus*	C6-C1 Claisen	Narigenin	3.21
**PKS3**	*Rubus ideaus*	Lactonization	coumaroyl triacetic acid lactone	3.21
**PKS4**	*Rubus ideaus*	C6-C1 Claisen	Narigenin	3.21
**PKS5**	*Rubus ideaus*	C6-C1 Claisen	Narigenin	3.21

		**FUNGAL ENZYMES**		
**CsyA**	*Aspergillus oryzae* RIB40	Lactonization	Alkylpyrones	3.21
**CsyB**	*Aspergillus oryzae* RIB40	Lactonization	Acetolated alkylpyrones	3.21
**ORAS**	*Neurospora crassa* OR74A	C2-C7 Aldol	Alkylresorcinols	3.21

The characterised type III PKSs used in this study are listed together with their main cyclization mechanism, major product group, and position in the phylogenetic tree (Figure 3).

Supplementary Table 4. List of best-fitting crystal structures used for cyanobacterial type III PKS protein modelling

Organism (type III PKS name)	Best fitting model	PDB number	TM-Score	C-Score
Azotobacter vinelandii ca (ArsC)	Crystal structure elucidated (Satou, et al. 2013)	3VS8	-	-
Cyanobium LEGE06113 (HidC)	PKS11	4JAO	0.901	1.71
Cylindrospermum lichenforme UTEX 2014 B (CylI)	Type III PKS from *Citrus x microcarpa*	3WD7	0.930	1.85
Leptolyngbya sp. PCC 7375	4-coumaroyl-CoA Ligase::Stilbene synthase fusion	3TSY	0.906	1.81
Medicago sativa (CHS)	Crystal structure elucidated (Ferrer, et al. 1999)	3EUO	-	-
Microcystis aeruginosa PCC 7806	Type III PKS from *Citrus x microcarpa*	3WD7	0.917	1.77
Microcystis aeruginosa SPC777	Type III PKS from *Citrus x microcarpa*	3WD7	0.889	1.69
Mycobacgterium tuberculosis H37Rv (PKS11)	Crystal structure elucidated (Saxena, et al. 2003)	4JAO	-	-
Prochlorococcus sp. MIT 1306	Type III PKS (Steely1) from *Dictyostelium discoideum* AX4	2H84	0.902	1.80
Rivularia sp. PCC 7116	Type III PKS from *Citrus x microcarpa*	3WD7	0.828	1.72

The first column lists type III PKSs computationally analysed and their corresponding species of origin. The second column lists reference crystal structures used as scaffolds to model uncharacterised type III PKSs. The third column lists Protein Database (PDB) numbers for reference crystal structures. For PKS11, CHS and ArsC the crystal structure was used, PDB numbers of the crystal structures are given.

REFERENCES

Abe I, Takahashi Y, Morita H, Noguchi H. 2001. Benzalacetone synthase. A novel polyketide synthase that plays a crucial role in the biosynthesis of phenylbutanones in Rheum palmatum. Eur J Biochem 268:3354-3359.

Akiyama T, Shibuya M, Liu HM, Ebizuka Y. 1999. p-Coumaroyltriacetic acid synthase, a new homologue of chalcone synthase, from Hydrangea macrophylla var. thunbergii. Eur J Biochem 263:834-839.

Bangera MG, Thomashow LS. 1999. Identification and characterization of a gene cluster for synthesis of the polyketide antibiotic 2,4-diacetylphloroglucinol from Pseudomonas fluorescens Q2-87. J Bacteriol 181:3155-3163.

Blin K, Shaw S, Steinke K, Villebro R, Ziemert N, Lee SY, Medema MH, Weber T. 2019. antiSMASH 5.0: updates to the secondary metabolite genome mining pipeline. Nucleic Acids Res 47:W81-W87.

Chen H, Tseng CC, Hubbard BK, Walsh CT. 2001. Glycopeptide antibiotic biosynthesis: Enzymatic assembly of the dedicated amino acid monomer (S)-3,5-dihydroxyphenylglycine. Proc Natl Acad Sci U S A 98:14901-14906.

Chen R, Zhang Q, Tan B, Zheng L, Li H, Zhu Y, Zhang C. 2017. Genome mining and activation of a silent PKS/NRPS gene cluster direct the production of totopotensamides. Org Lett 19:5697-5700.

Cortés J, Velasco J, Foster G, Blackaby AP, Rudd BAM, Wilkinson B. 2002. Identification and cloning of a type III polyketide synthase required for diffusible pigment biosynthesis in Saccharopolyspora erythraea. Mol Microbiol 44:1213-1224.

Costa M, Sampaio-Dias IE, Castelo-Branco R, Scharfenstein H, Rezende de Castro R, Silva A, Schneider MPC, Araújo MJ, Martins R, Domingues VF, et al. 2019. Structure of hierridin C, synthesis of hierridins B and C, and evidence for prevalent alkylresorcinol biosynthesis in picocyanobacteria. J Nat Prod 82:393-402.

Edgar RC. 2004. MUSCLE: multiple sequence alignment with high accuracy and high throughput. Nucleic Acids Res 32:1792-1797.

Ferrer JL, Jez JM, Bowman ME, Dixon RA, Noel JP. 1999. Structure of chalcone synthase and the molecular basis of plant polyketide biosynthesis. Nat Struct Biol 6:775-784.

Frangeul L, Quillardet P, Castets AM, Humbert JF, Matthijs HC, Cortez D, Tolonen A, Zhang CC, Gribaldo S, Kehr JC, et al. 2008. Highly plastic genome of *Microcystis aeruginosa* PCC 7806, a ubiquitous toxic freshwater cyanobacterium. BMC Genomics 9:274.

Funa N, Awakawa T, Horinouchi S. 2007. Pentaketide resorcylic acid synthesis by type III polyketide synthase from *Neurospora crassa*. J Biol Chem 282:14476-14481.

Funa N, Ohnishi Y, Ebizuka Y, Horinouchi S. 2002. Alteration of reaction and substrate specificity of a bacterial type III polyketide synthase by site-directed mutagenisis. Biochem J 367:781-789.

Funa N, Ozawa H, Hirata A, Horinouchi S. 2006. Phenolic lipid synthesis by type III polyketide synthases is essential for cyst formation in *Azotobacter vinelandii*. Proc Natl Acad Sci U S A 103:6356-6361.

Funabashi M, Funa N, Horinouchi S. 2008. Phenolic lipids synthesized by type III polyketide synthase confer penicillin resistance on *Streptomyces griseus*. J Biol Chem 283:13983-13991.

Ghimire GP, Oh TJ, Liou K, Sohng JK. 2008. Identification of a cryptic type III polyketide synthase (1,3,6,8-tetrahydroxynaphthalene synthase) from *Streptomyces peucetius* ATCC 27952. Mol Cells 26:362-367.

Gilchrist CLM, Chooi YH. 2021. Clinker and clustermap.js: Automatic generation of gene cluster comparison figures. Bioinformatics btab007, https://doi.org/10.1093/bioinformatics/btab007.

Gruschow S, Buchholz TJ, Seufert W, Dordick JS, Sherman DH. 2007. Substrate profile analysis and ACP-mediated acyl transfer in Streptomyces coelicolor Type III polyketide synthases. Chembiochem 8:863-868.

Hashimoto M, Ishida S, Seshime Y, Kitamoto K, Fujii I. 2013. *Aspergillus oryzae* type III polyketide synthase CsyB uses a fatty acyl starter for the biosynthesis of csypyrone B compounds. Bioorg Med Chem Lett 23:5637-5640.

Izumikawa M, Shipley PR, Hopke JN, O'Hare T, Xiang L, Noel JP, Moore BS. 2003. Expression and characterization of the type III polyketide synthase 1,3,6,8-tetrahydroxynaphthalene synthase from *Streptomyces coelicolor* A3(2). J Ind Microbiol Biotechnol 30:510-515.

Jez JM, Ferrer JL, Bowman ME, Dixon RA, Noel JP. 2000. Dissection of malonyl-coenzyme A decarboxylation from polyketide formation in the reaction mechanism of a plant polyketide synthase. Biochemistry 39:890-902.

May DS, Chen WL, Lantvit DD, Zhang X, Krunic A, Burdette JE, Eustaquio A, Orjala J. 2017. Merocyclophanes C and D from the cultured freshwater cyanobacterium *Nostoc* sp. (UIC 10110). J Nat Prod 80:1073-1080.

McKhann HI, Hirsch AM. 1994. Isolation of chalcone synthase and chalcone isomerase cDNAs from alfalfa (*Medicago sativa* L.): highest transcript levels occur in young roots and root tips. Plant Mol Biol 24:767-777.

Mizuuchi Y, Shi SP, Wanibuchi K, Kojima A, Morita H, Noguchi H, Abe I. 2009. Novel type III polyketide synthases from *Aloe arborescens*. FEBS Journal 276:2391-2401.

Mizuuchi Y, Shimokawa Y, Wanibuchi K, Noguchi H, Abe I. 2008. Structure function analysis of novel type III polyketide synthases from *Arabidopsis thaliana*. Biol Pharm Bull 31:2205-2210.

Moriguchi T, Kita M, Tomono Y, EndoInagaki T, Omura M. 1999. One type of chalcone synthase gene expressed during embryogenesis regulates the flavonoid accumulation in citrus cell cultures. Plant Cell Physiol 40:651-655.

Nakamura H, Hamer HA, Sirasani G, Balskus EP. 2012. Cylindrocyclophane biosynthesis involves functionalization of an unactivated carbon center. J Am Chem Soc 134:18518-18521.
Nakano C, Ozawa H, Akanuma G, Funa N, Horinouchi S. 2009. Biosynthesis of aliphatic polyketides by type III polyketide synthase and methyltransferase in *Bacillus subtilis*. J Bacteriol 191:4916-4923.
Navarro-Munoz JC, Selem-Mojica N, Mullowney MW, Kautsar SA, Tryon JH, Parkinson EI, De Los Santos ELC, Yeong M, Cruz-Morales P, Abubucker S, et al. 2020. A computational framework to explore large-scale biosynthetic diversity. Nat Chem Biol 16:60-68.
Nualkaew N, Morita H, Shimokawa Y, Kinjo K, Kushiro T, De-Eknamkul W, Ebizuka Y, Abe I. 2012. Benzophenone synthase from *Garcinia mangostana* L. pericarps. Phytochemistry 77:60-69.
Parvez A, Giri S, Giri GR, Kumari M, Bisht R, Saxena P. 2018. Novel type III polyketide synthases biosynthesize methylated polyketides in *Mycobacterium marinum*. Sci Rep 8:6529.
Pfeifer V, Nicholson GJ, Ries J, Recktenwald J, Schefer AB, Shawky RM, Schröder J, Wohlleben W, Pelzer S. 2001. A polyketide synthase in glycopeptide biosynthesis: the biosynthesis of the non-proteinogenic amino acid (S)-3,5-dihydroxyphenylglycine. J Biol Chem 276:38370-38377.
Pootoolal J, Thomas MG, Marshall CG, Neu JM, Hubbard BK, Walsh CT, Wright GD. 2002. Assembling the glycopeptide antibiotic scaffold: The biosynthesis of A47934 from *Streptomyces toyocaensis* NRRL15009. Proc Natl Acad Sci U S A 99:8962-8967.
Preisitsch M, Heiden SE, Beerbaum M, Niedermeyer TH, Schneefeld M, Herrmann J, Kumpfmuller J, Thurmer A, Neidhardt I, Wiesner C, et al. 2016. Effects of halide ions on the carbamidocyclophane biosynthesis in *Nostoc* sp. CAVN2. Mar Drugs 14:21.
Ronquist F, Teslenko M, van der Mark P, Ayres DL, Darling A, Höhna S, Larget B, Liu L, Suchard MA, Huelsenbeck JP. 2012. MrBayes 3.2: efficient Bayesian phylogenetic inference and model choice across a large model space. Syst Biol 61:539-542.
Satou R, Miyanaga A, Ozawa H, Funa N, Katsuyama Y, Miyazono K, Tanokura M, Ohnishi Y, Horinouchi S. 2013. Structural basis for cyclization specificity of two *Azotobacter* type III polyketide synthases: a single amino acid substitution reverses their cyclization specificity. J Biol Chem 288:34146-34157.
Saxena P, Yadav G, Mohanty D, Gokhale RS. 2003. A new family of type III polyketide synthases in *Mycobacterium tuberculosis*. J Biol Chem 278:44780-44790.
Schanz S, Schroder G, Schroder J. 1992. Stilbene synthase from Scots pine (*Pinus sylvestris*). FEBS Lett 313:71-74.
Seshime Y, Juvvadi PR, Kitamoto K, Ebizuka Y, Nonaka T, Fujii I. 2010. *Aspergillus oryzae* type III polyketide synthase CsyA is involved in the biosynthesis of 3,5-dihydroxybenzoic acid. Bioorg Med Chem Lett 20:4785-4788.
Sirakova TD, Dubey VS, Cynamon MH, Kolattukudy PE. 2003. Attenuation of *Mycobacterium tuberculosis* by disruption of a *mas*-like gene or a chalcone synthase-like gene, which causes deficiency in dimycocerosyl phthiocerol synthesis. J Bacteriol 185:2999-3008.
Takeuchi A, Matsumoto S, Hayatsu M. 1994. Chalcone synthase from *Camellia sinensis*: isolation of the cDNAs and the organ-specific and sugar-responsive expression of the genes. Plant Cell Physiol 35:1011-1018.
Taura F, Tanaka S, Taguchi C, Fukamizu T, Tanaka H, Shoyama Y, Morimoto S. 2009. Characterization of olivetol synthase, a polyketide synthase putatively involved in cannabinoid biosynthetic pathway. FEBS Lett 583:2061-2066.
Thanapipatsiri A, Gomez-Escribano JP, Song L, Bibb MJ, Al-Bassam M, Chandra G, Thamchaipenet A, Challis GL, Bibb MJ. 2016. Discovery of unusual biaryl polyketides by activation of a silent *Streptomyces venezuelae* biosynthetic gene cluster. ChemBioChem 17:2189-2198.
Ueda K, Kim KM, Beppu T, Horinouchi S. 1995. Overexpression of a gene cluster encoding a chalcone synthase-like protein confers redbrown pigment production in *Streptomyces griseus*. J Antibiot (Tokyo) 48:638-646.
Wenzel SC, Bode HB, Kochems I, Muller R. 2008. A type I/type III polyketide synthase hybrid biosynthetic pathway for the structurally unique ansa compound kendomycin. Chembiochem 9:2711-2721.
Zeng J, Decker R, Zhan J. 2012. Biochemical characterization of a type III polyketide biosynthetic gene cluster from *Streptomyces toxytricini*. Appl Biochem Biotechnol 166:1020-1033.
Zheng D, Schröder G, Schröder J, Hrazdina G. 2001. Molecular and biochemical characterization of three aromatic polyketide synthase genes from *Rubus idaeus*. Plant Mol Biol 46:1-15.
